# Lonafarnib and everolimus reduce pathology in iPSC-derived tissue engineered blood vessel model of Hutchinson-Gilford Progeria Syndrome

**DOI:** 10.1038/s41598-023-32035-3

**Published:** 2023-03-28

**Authors:** Nadia O. Abutaleb, Leigh Atchison, Leandro Choi, Akhil Bedapudi, Kevin Shores, Yantenew Gete, Kan Cao, George A. Truskey

**Affiliations:** 1 Department of Biomedical Engineering, Duke University, Durham, NC USA; 2 Department of Cell Biology and Molecular Genetics, University of Maryland, College Park, MD USA

**Keywords:** Diseases, Cardiovascular diseases, Vascular diseases, Biotechnology, Regenerative medicine, Tissue engineering

## Abstract

Hutchinson-Gilford Progeria Syndrome (HGPS) is a rare, fatal genetic disease that accelerates atherosclerosis. With a limited pool of HGPS patients, clinical trials face unique challenges and require reliable preclinical testing. We previously reported a 3D tissue engineered blood vessel (TEBV) microphysiological system fabricated with iPSC-derived vascular cells from HGPS patients. HGPS TEBVs exhibit features of HGPS atherosclerosis including loss of smooth muscle cells, reduced vasoactivity, excess extracellular matrix (ECM) deposition, inflammatory marker expression, and calcification. We tested the effects of HGPS therapeutics Lonafarnib and Everolimus separately and together, currently in Phase I/II clinical trial, on HGPS TEBVs. Everolimus decreased reactive oxygen species levels, increased proliferation, reduced DNA damage in HGPS vascular cells, and improved vasoconstriction in HGPS TEBVs. Lonafarnib improved shear stress response of HGPS iPSC-derived endothelial cells (viECs) and reduced ECM deposition, inflammation, and calcification in HGPS TEBVs. Combination treatment with Lonafarnib and Everolimus produced additional benefits such as improved endothelial and smooth muscle marker expression and reduced apoptosis, as well as increased TEBV vasoconstriction and vasodilation. These results suggest that a combined trial of both drugs may provide cardiovascular benefits beyond Lonafarnib, if the Everolimus dose can be tolerated.

## Introduction

Hutchinson-Gilford Progeria Syndrome (HGPS) is a rare, fatal genetic disease that accelerates atherosclerosis in children^1^. It is caused by a de novo point mutation in the LMNA gene that results in production of progerin, an aberrantly spliced version of prelamin A that remains permanently farnesylated and methylated at its C-terminus^1^. The farnesyl group anchors progerin to the nuclear lamina where it accumulates and causes DNA repair defects, altered gene expression, disorganized heterochromatin, telomere shortening, and cell senescence^1,2^. Patient symptoms include subcutaneous fat loss, progressive joint contracture, alopecia, bony dysplasia, and failure to thrive^1,3^. Fatality is caused by progressive atherosclerosis leading to heart attack or stroke by 15 years of age^4^.

Many HGPS children exhibit hypertension and altered ankle-brachial index, similar to that found in atherosclerosis of older non-HGPS individuals, although endothelial induced vasodilation is normal^5^. While the atherosclerotic lesions are similar to those found in older, non-HGPS individuals with atherosclerosis, in HGPS, cholesterol does not accumulate and adventitial thickening and fibrosis are observed^4,6^. Vascular SMC loss occurs throughout the HGPS vessel wall as SMCs are replaced by a dense matrix of proteoglycans and collagen, resulting in vascular stiffening that causes hypertension^4,6^. HGPS fibrotic plaques incorporate excessively expressed ECM proteins such as collagens I, III, IV, V, and VI^7^.

With a limited pool of ~ 350 HGPS patients worldwide, testing potential therapeutics for the disease faces unique and significant challenges^8^. Only one or two new approaches can be studied at a time, clinical trials require global recruitment but still suffer from low subject numbers, and historical controls must be used without placebo groups^8^. These challenges drive the need for accurate in vitro models that offer reliable preclinical testing of potential therapeutics before they progress into clinical trials. To date, in vitro HGPS preclinical studies rely on 2D cell culture which does not accurately model the 3D physiological microenvironment. Further, very few HGPS murine models exhibit cardiovascular pathology which appears after many months^9^. Since atherosclerosis is the fatal symptom of HGPS, a biomimetic model of HGPS cardiovascular pathology is an ideal platform for preclinical testing of therapeutics. Preclinical testing models that accurately predict drug responses can better identify candidates for clinical trials and determine the maximum tolerated dose (MTD) of therapeutics to potentially avoid toxicities that commonly occur during the initial stages of clinical trials.

To provide a human cell in vitro vascular model of HGPS, we developed 3D tissue engineered blood vessels (TEBVs) using induced pluripotent stem cell (iPSC)-derived vascular smooth muscle cells (viSMCs) with a luminal monolayer of iPSC-derived endothelial cells (viECs)^10^. TEBVs are fabricated within a few hours and perfused at physiological shear stress for several weeks. We differentiated iPSCs from healthy and HGPS patients into endothelial cells and smooth muscle cells that exhibit key markers and functional features of these cells. We then used the HGPS cells to create an HGPS TEBV model that replicates vascular disease characteristics after 3 weeks, including progerin expression, vascular calcification, loss of medial smooth muscle cells, reduced vasoactivity, and excess extracellular matrix (ECM) deposition^10^. Our model indicated a specific role of the endothelium in disease progression as viECs from HGPS patients exhibited blunted response to physiological shear stress and inflammatory marker expression^10^. Further, TEBVs can model early steps in atherosclerosis in straight^11^ and branched vessels^12^. This patient-specific 3D tissue engineered blood vessel platform offers the opportunity to study disease mechanisms and evaluate therapeutics in a controlled, biomimetic microenvironment.

Permanent farnesylation of progerin anchors the protein to the nuclear lamina, causing nuclear blebbing or misshapen nuclei, thickened nuclear lamina, and impaired mechanotransduction^13^. Cell and mouse models of HGPS have indicated that the farnesyl group of progerin is partly responsible for the HGPS phenotype^1,14^. Lonafarnib is a farnesyltransferase inhibitor (FTI) that inhibits post-translational farnesylation of progerin, reducing progerin intercalation into the nuclear envelope^1,15,16^. FTI treatment improves nuclear morphology in HGPS cells^17–20^, extends lifespan, improves skeletal properties, increases body weight and adipose tissue, and improves cardiovascular phenotype in animal models^2,21–24^. A clinical trial testing Lonafarnib indicated treatment increased body weight, reduced cardiovascular stiffness, increased bone density, and decreased mortality of trial participants^3,15,25^. Lonafarnib is currently the only FDA-approved therapy for HGPS.

There is currently a Phase I/II clinical trial to test the combination of Lonafarnib and the rapamycin analog Everolimus for HGPS (ClinicalTrials.gov Identifier: NCT02579044). Treatment of HGPS cells with rapamycin or its analogues increases autophagy-mediated progerin clearance, resulting in reduced nuclear abnormalities, senescence, and DNA damage levels in HGPS fibroblasts^26,27^. While rapamycin or its analogues have not been used in animal models of HGPS, genetic reduction of mTOR in a mouse progeria model improved lifespan, although progerin remained in cells^28^. Treating HGPS patients with the two drugs might enhance the effectiveness of Lonafarnib, although there are no published studies testing Lonafarnib and rapamycin combination treatment in HGPS mouse models. The objective of this study was to test the effect of Lonafarnib and Everolimus, separately and together, to assess if the beneficial effects of the drug combination on HGPS TEBV structure and function. Results of this study might inform clinical trial results.

## Results

### Everolimus treatment reduces progerin expression and misshapen nuclei in HGPS viSMCs and viECs

Treatment of viSMCs and viECs for 1 week with either 1 µM Lonafarnib or 0.1 µM Everolimus leads to minimal cell death and high cell viability, comparable to untreated and vehicle controls (Fig. S1). In SMCs, treatment with 1 µM Lonafarnib and 0.05 µM Everolimus does not reduce cell viability, while doubling the dose to 2 µM Lonafarnib and 0.1 µM Everolimus significantly decreases cell viability. For subsequent experiments, cells and TEBVs were treated with 1 µM Lonafarnib alone, 0.1 µM Everolimus alone, or 1 µM Lonafarnib combined with 0.05 µM Everolimus.

**Figure 1:**
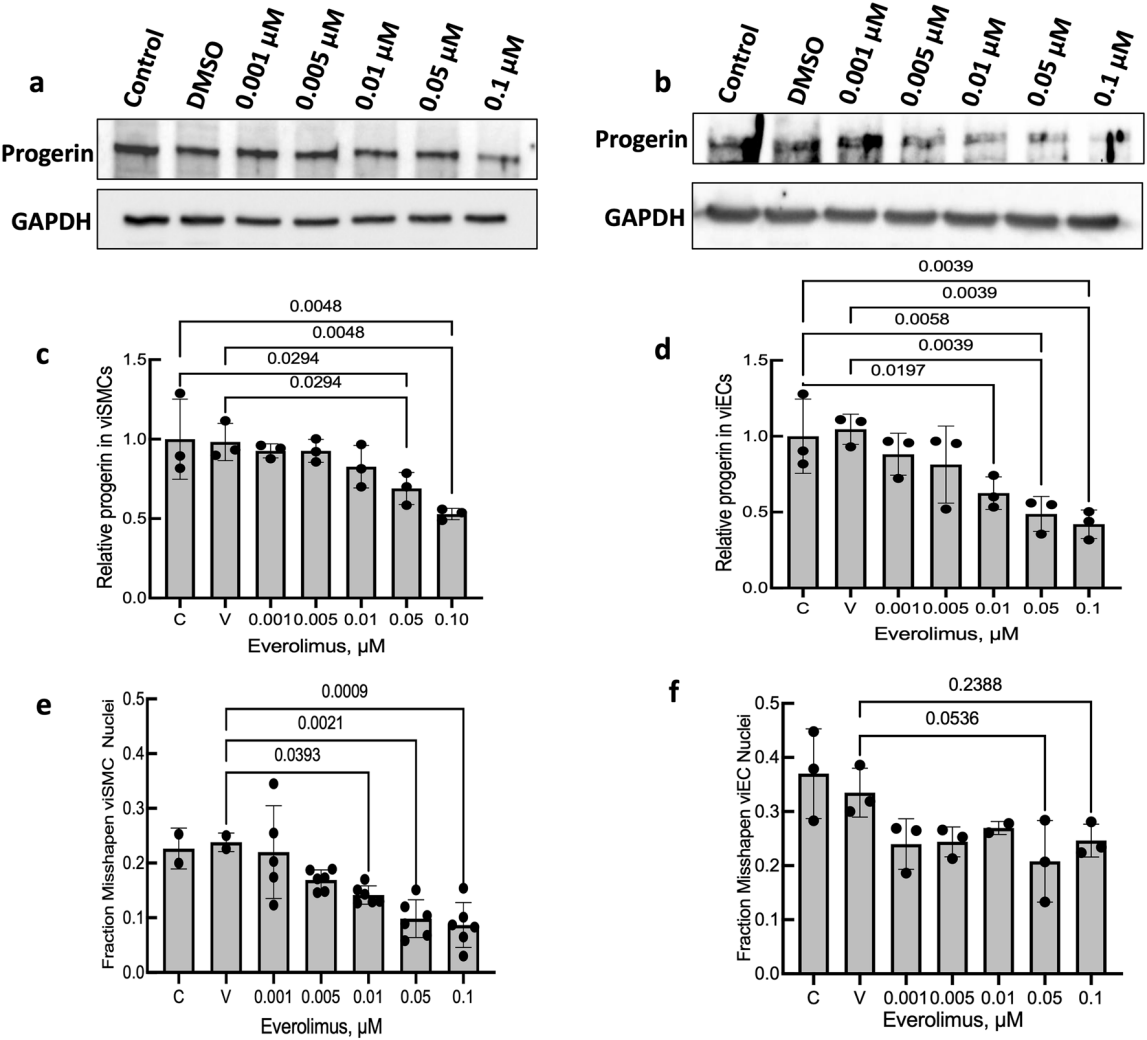
Everolimus reduces progerin expression and misshapen nuclei in HGPS viSMCs and viECs. (**a**,** b**) Cropped images of Western blot gels of progerin and (**c**, **d**) quantification in HGPS 167 CL2 viSMCs (**a**) and viECs (**b**) treated with Everolimus (Ev). Entire gels for each of the experiments performed with viSMCs and viECs are shown in Fig. S2. Data presented as fold change progerin expression compared with untreated HGPS control. ‘V’ indicates DMSO vehicle control. (**e, f**) Misshapen nuclei in HGPS 167 CL2 viSMCs (**e**) and viECs (**f**) treated with Everolimus for 7 days. Representative images are shown in Fig. S4. ‘V’ indicates DMSO vehicle control. Data presented as mean ± SD. N = 2–6 experiments per group.

Next, progerin levels were measured after treating viSMCs and viECs for 7 days with 0.001–0.1 µM Everolimus, based on prior published studies^29^ and our own initial testing (Fig. 1a,b). Quantifying Western blots (Fig. 1c,d) indicated that by 0.05 µM Everolimus, progerin levels were significantly below vehicle levels. (The entire individual Western blots are shown in Fig. S2).

Rapamycin treatment of HGPS fibroblasts does not affect lamin A/C^29^. Likewise, using a lamin A/C antibody, we found that one-week treatment of viSMCs or viECs with 0.1 µM Everolimus did not cause a statistically significant decline in lamin A or lamin C in viSMCs (Fig. S3a,b), but did reduce lamin C in viECs (Fig. S3c,d). This antibody also recognizes progerin. After treatment of viSMCs with 0.1 µM Everolimus progerin decreased relative to untreated or DMSO treated viSMCs, as was seen with the progerin-specific antibody (Fig. 1a,c). In viECs, Everolimus caused a decline in progerin, but progerin declined after DSMO treatment, which differed from the anti-progerin antibody (Fig. 1b,d).

Nuclei shape in HGPS is distorted, often showing blebs or non-elliptical shape^30^ (Fig. S4). We previously showed that HGPS viSMCs and viECs exhibit a higher fraction of misshapen nuclei than healthy cells^10^. iPSC-derived HGPS viSMCs and viECs treated with Everolimus for 7 days exhibited reduced levels of misshapen nuclei compared with no treatment and vehicle controls, reaching significance at 0.05 µM in both viSMCs and viECs (Fig. 1e,f).

### Everolimus reduces ROS levels, increases proliferation, and decreases DNA damage in HGPS viSMCs and viECs

**Figure 2:**
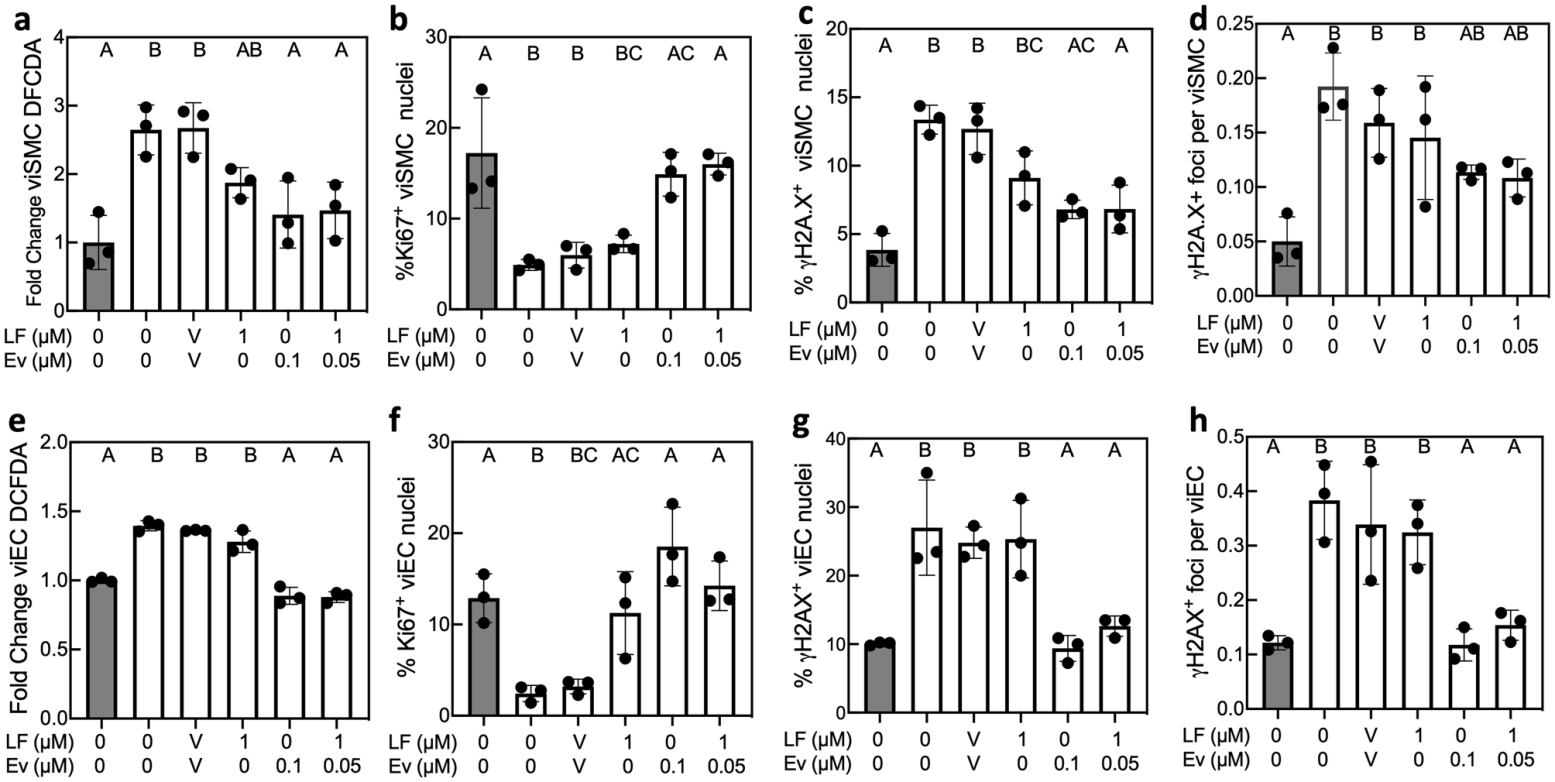
Everolimus reduces ROS levels, increases proliferation, and decreases DNA damage in HGPS 167 CL2 viSMCs and viECs. Healthy viSMCs (**a**–**d**) and viECs (**e**–**h**) and HGPS 167 CL2 viSMCs and viECs were treated with different combinations of Lonafarnib (LF) and Everolimus (Ev). ‘V’ indicates DMSO vehicle control. viECs and viSMCs from healthy donors are shown as gray bars and HGPS viECs and viSMCs are shown as white bars. Fold change of DCFDA mean fluorescence intensity compared with healthy in viSMCs (**a**) and viECs (**e**). Percent positive Ki67 nuclei in viSMCs (**b**) and viECs (**f**). Percent nuclei with ɣH2A.X foci in viSMCs (**c**) and viECs (**g**). Number of ɣH2A.X foci per total cells in viSMCs (**d**) and viECs (**h**). Data presented as mean ± SD. N = 3 experiments per group. Groups connected by the same letter are not significantly different from each other (p > 0.05). Groups labeled with different letters are significantly different from each other (p < 0.05). Exact p-values for significant differences are provided in Table S1.

HGPS viSMCs from two donors (167 CL2 and 003 CL1D) exhibited significantly increased ROS levels, indicated by higher level of DCFDA mean fluorescence intensity, and reduced proliferation, marked by decreased Ki67 expression, compared with healthy viSMCs (Fig. 2a,b for 167 CL2 and Fig. S5a,b for 003 CL1D). HGPS viSMCs had significantly increased levels of DNA double-stranded breaks (DSBs), marked by a higher percentage of nuclei staining positively for ɣH2A.X foci (Fig. 2c, Fig. S5c) and more ɣH2A.X foci per cell (Fig. 2d, Fig. S5d). viECs from both HGPS donors displayed similar results with higher ROS levels (Fig. 2e, Fig. S5e), reduced proliferation (Fig. 2f, Fig. S5f), and more DNA DSBs (Fig. 2g,h, Fig. S5g,h). Representative images of DCFDA, Ki67, and γH2A.X staining are shown in Figs. S6, S7, and S8.

For viSMCs from both HGPS donors, Lonafarnib partially reduced ROS levels, but to a level not significantly different from untreated HGPS or vehicle control (Fig. 2a, Fig. S5a). In contrast, Everolimus treatment reduced ROS to a level not significantly different than healthy viSMCs (Fig. 2a, Fig. S5a). Lonafarnib treatment did not improve HGPS viSMC proliferation for either donor (Fig. 2b, Fig. S5b). Everolimus and combination treatments significantly improved proliferation in HGPS viSMCs from donor 167 (Fig. 2b) but not in the 003 donor, which was at a higher level without drug treatment (Fig. S5b). Lonafarnib reduced the percentage of nuclei with DNA DSBs in viSMCs from donor 003 (Fig. S5c) but not in viSMCs from donor 167 (Fig. 2c). Everolimus treatment significantly reduced the percentage of nuclei with DNA DSBs to healthy levels in viSMCs from both HGPS donors (Fig. 2c, Fig. S5c).

In viECs, Lonafarnib partially reduced ROS levels in only the 003 line (Fig. 2e, Fig. S5e). Everolimus reduced ROS in HGPS viECs from both donors to a level that was not significantly different than healthy cells (Fig. 2e, Fig. S5e). Combination treatment with Lonafarnib and Everolimus reduced ROS levels to healthy levels in viECs from both donors (Fig. 2e, Fig. S5e). Lonafarnib only improved viEC proliferation in donor 167 (Fig. 2f). Everolimus and combination treatment significantly improved proliferation in viECs from both HGPS donors (Fig. 2f, Fig. S5f). In viECs, Lonafarnib had no significant effect on DNA DSBs for either donor (Fig. 2g,h, Fig. S5g,h). In contrast, Everolimus reduced percentage of nuclei with DNA DSBs and DNA DSBs per nucleus in viECs from both donors (Fig. 2g,h, Fig. S5g,h). Combination treatment resulted in similar DNA DSB as treatment with Everolimus alone in viECs from both donors (Fig. 2g,h, Fig. S5g,h).

### Lonafarnib and Everolimus improve NO production and shear stress-sensitive gene expression in viECs exposed to physiological shear stress

**Figure 3:**
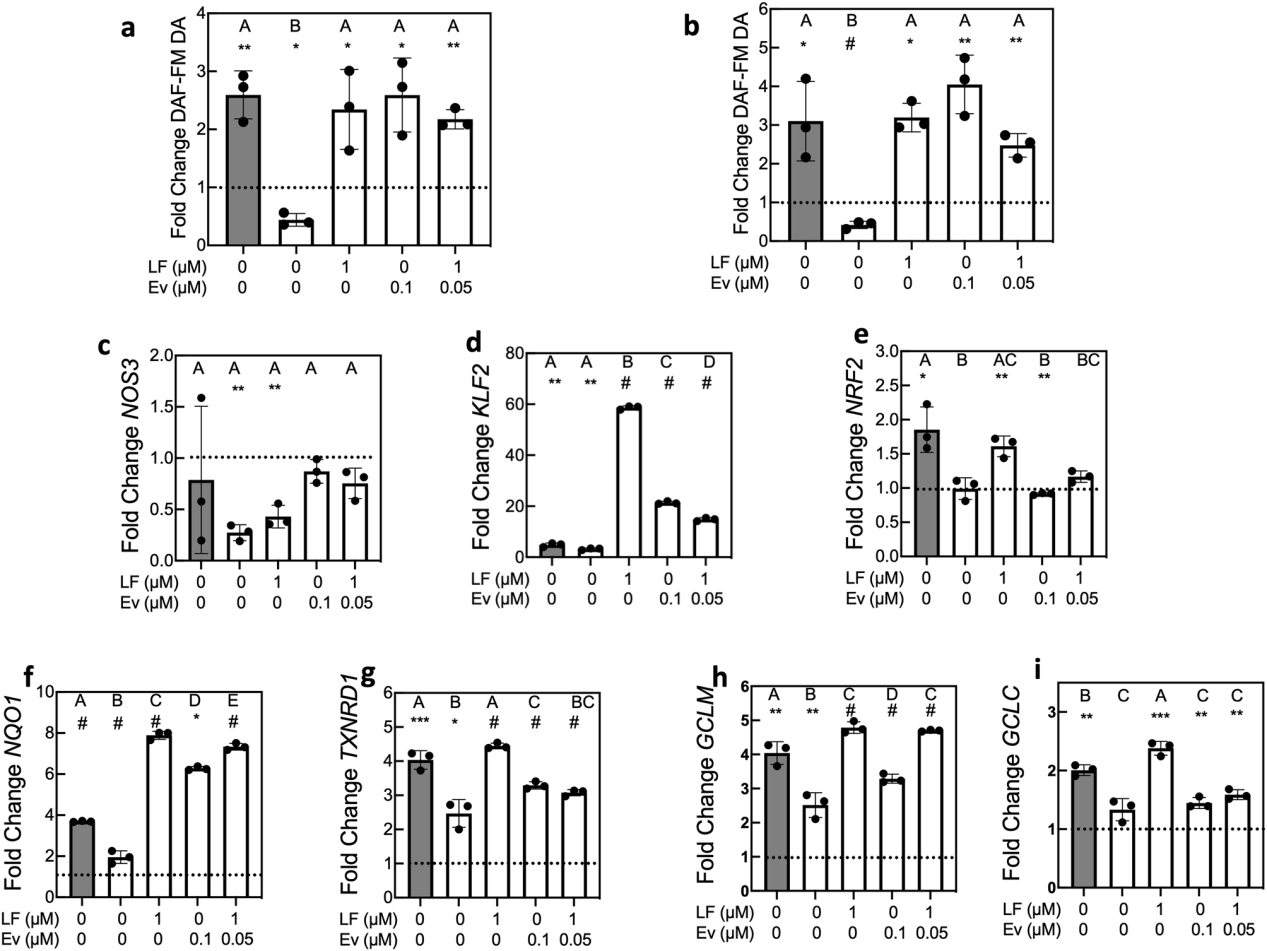
Lonafarnib and Everolimus improve NO production and flow-mediated gene expression in HGPS viECs exposed to physiological shear stress. (**a**, **b**) DAF-FM diacetate (DA) mean fluorescence intensity (MFI) in healthy (gray), HGPS 167 CL2 (**a**), and HGPS 003 CL1D (**b**) viECs exposed to 12 dynes/cm^2^ for 24 h. Data presented as fold change DAF-FM DA MFI under shear stress compared with static condition from the same group. Gray bar represents healthy viECs. (**c**-**i**) Gene expression of (**c**) *NOS3*, (**d**) *KLF2*, (**e**) *NRF2*, (**f**) *NQO1*, (**g**) *TXNRD1*, (**h**) *GCLM,* and (**i**) *GCLC* in HGPS 167 CL2 viECs exposed to 12 dynes/cm^2^. Data were normalized to *GAPDH* expression and gene expression in viECs exposed to shear stress was normalized to respective viECs under static culture from the same group. Dashed line indicates normalized static control. Data presented as mean ± SD. N = 3 experiments per group. Asterisks indicate significant differences between each shear stress value and its own static control: *p < 0.05, **p < 0.01, ***p < 0.001, ^#^p < 0.0001. Letters indicate significant differences between treatment groups under shear stress: groups labeled with different letters are significantly different from each other (p < 0.05). Exact p-values for significant differences for comparisons between treatment groups under shear stress are provided in Table S2.

To test the response of healthy and HGPS viECs to physiological shear stress which establishes normal endothelial cell function in vivo, we applied 12 dynes/cm^2^ shear stress for 24 h after 6-day static culture with or without treatment. When used, the drugs were applied during the 6-day static period and during the 24-h flow period. As marked by an increase in the relative DAF-FM diacetate mean fluorescence intensity, healthy viECs produced higher nitric oxide (NO) levels when exposed to shear stress compared with the same viECs under static conditions (Fig. 3a,b). In contrast, HGPS viECs from both donors exhibited reduced levels of NO production after exposure to shear stress, consistent with a dysfunctional endothelial phenotype (Fig. 3a,b)^31^. Lonafarnib treatment, Everolimus treatment, and combination treatment all restored NO production under shear stress to healthy levels in HGPS viECs from both donors (Fig. 3a,b). (Representative images are shown in Fig. S9).

We tested the expression of several shear stress-sensitive genes in healthy viECs and HGPS viECs with or without Lonafarnib and Everolimus. NO production is regulated by the expression of endothelial NO synthase which is encoded in the *NOS3* gene. *KLF2* (Kruppel-like factor 2) is a major anti-inflammatory and anti-thrombotic flow-responsive transcription factor which is upregulated by EC exposure to laminar flow and regulates ∼ 46% of EC flow-sensitive genes. *KLF2* activates antioxidant transcription factor nuclear- erythroid 2-related factor 2 *(NRF2)* and both of these genes promote EC quiescence^32^. Since NRF2 transcription and nuclear location are affected by progerin^33^, we evaluated several *NRF2* downstream targets: NAD(P)H-quinone oxidoreductase 1 (*NQO1*), thioredoxin reductase 1 (*TXNRD1*), glutamate-cysteine ligase modifier subunit (*GCLM*), and glutamate-cysteine ligase catalytic subunit *(GCLC)*^34^. While *NOS3* expression did not significantly change in healthy viECs exposed to shear stress compared with viECs under static conditions, the expression of *KLF2*, *NRF2*, and *NRF2* targets *NQO1*, *TXNRD1*, *GCLM*, and *GCLC* were upregulated (Fig. 3c–i). In contrast, after being exposed to 12 dynes/cm^2^ for 24 h, HGPS viECs from donor 167 downregulated *NOS3* (Fig. 3c) while *NRF2* and *GCLC* expression did not significantly change (Fig. 3e,i). *NQO1, TXNRD1,* and *GCLM* were upregulated by HGPS viECs to a significantly lesser extent than healthy viECs under the same shear stress conditions (Fig. 3f–h). *KLF2* expression in HGPS viECs was reduced compared with healthy viECs but was not statistically significant (p = 0.0503, Fig. 3d).

Lonafarnib treatment did not affect *NOS3* expression by HGPS viECs (Fig. 3c). In HGPS viECs treated with Everolimus and combination treatment, *NOS3* was restored to a level not significantly different from static culture, similar to healthy viECs (Fig. 3c). Lonafarnib did significantly increase expression of *KLF2*, *NRF2*, *NQO1*, *TXNRD1*, *GCLM*, and *GCLC* in HGPS viECs from donor 167 to levels similar to or higher than healthy expression levels (Fig. 3d–i). Everolimus significantly increased expression of *KLF2, NQO1, TXNRD1,* and *GCLM* but to levels significantly lower than Lonafarnib treatment (Fig. 3d,f–h). Everolimus treatment had no significant effect on *NRF2* or *GCLC* expression (Fig. 3e,i). Combination treatment did not offer any additional improvement over Lonafarnib monotherapy for *KLF2, NRF2, NQO1, TXNRD1, GCLM,* or *GCLC*.

### Lonafarnib and Everolimus improve HGPS TEBV function and expression of SMC and EC markers

**Figure 4:**
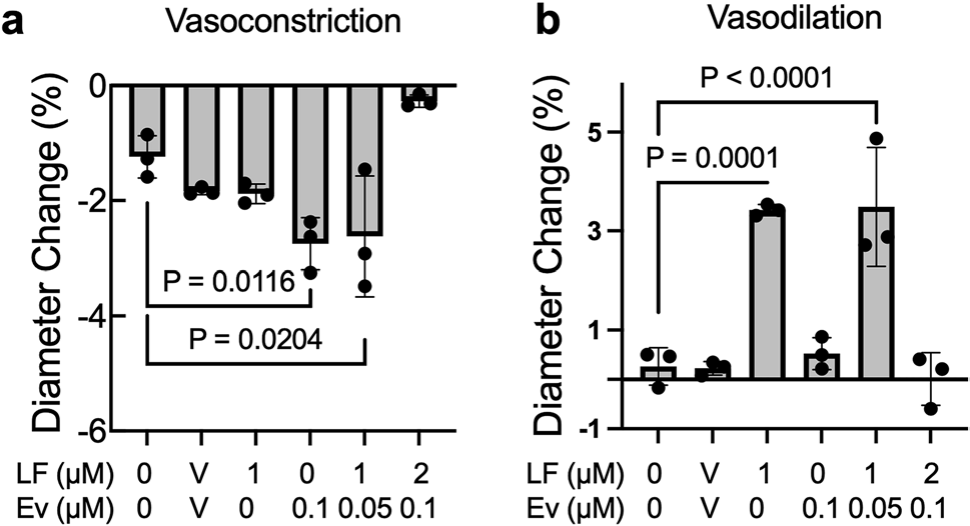
Lonafarnib and Everolimus treatment improves HGPS TEBV vasoactivity. HGPS 167 CL2 TEBVs were matured for 3 weeks, treated with Lonafarnib (LF) and Everolimus (Ev) for 1 week, then exposed to 1 µM phenylephrine followed by 1 µM acetylcholine for 5 min each and diameter change measured. Data presented as mean ± SD. N = 3 experiments per group. Results are compared to the reference condition without Lonafarnib and Everolimus.

HGPS TEBVs were perfused for 3 weeks without treatment to develop a disease phenotype and then were left untreated or were treated with 1 µM Lonafarnib alone, 0.1 µM Everolimus alone, or a combination of 1 µM Lonafarnib and 0.05 µM Everolimus for one week. We previously showed that compared with TEBVs made with healthy cells, HGPS TEBVs exhibit significantly reduced vasoconstriction when exposed to 1 µM phenylephrine and significantly reduced vasodilation when exposed to 1 µM acetylcholine^10^. Lonafarnib treatment did not affect vasoconstriction in response to phenylephrine but resulted in significantly increased dilation in response to acetylcholine in HGPS TEBVs compared to untreated and vehicle controls (Fig. 4a,b). In contrast, HGPS TEBVs treated with Everolimus alone showed significantly greater constriction compared to untreated and vehicle controls, but no significant effect on dilation (Fig. 4a,b). Combination treatment with 1 µM Lonafarnib and 0.05 µM Everolimus showed a statistically significant additive effect upon dilation and constriction. The maximum level of vasoconstriction and vasodilation is comparable to TEBVs made with viECs and viSMCs from non-HGPS donors^10^. Treatment with the toxic combination dose of 2 µM Lonafarnib and 0.1 µM Everolimus did not improve vasodilation or vasoconstriction. TEBV treatment with Lonafarnib or Everolimus for two weeks did not offer any additional benefit in vasoactivity over 1-week treatment (Fig. S10a,b).

**Figure 5:**
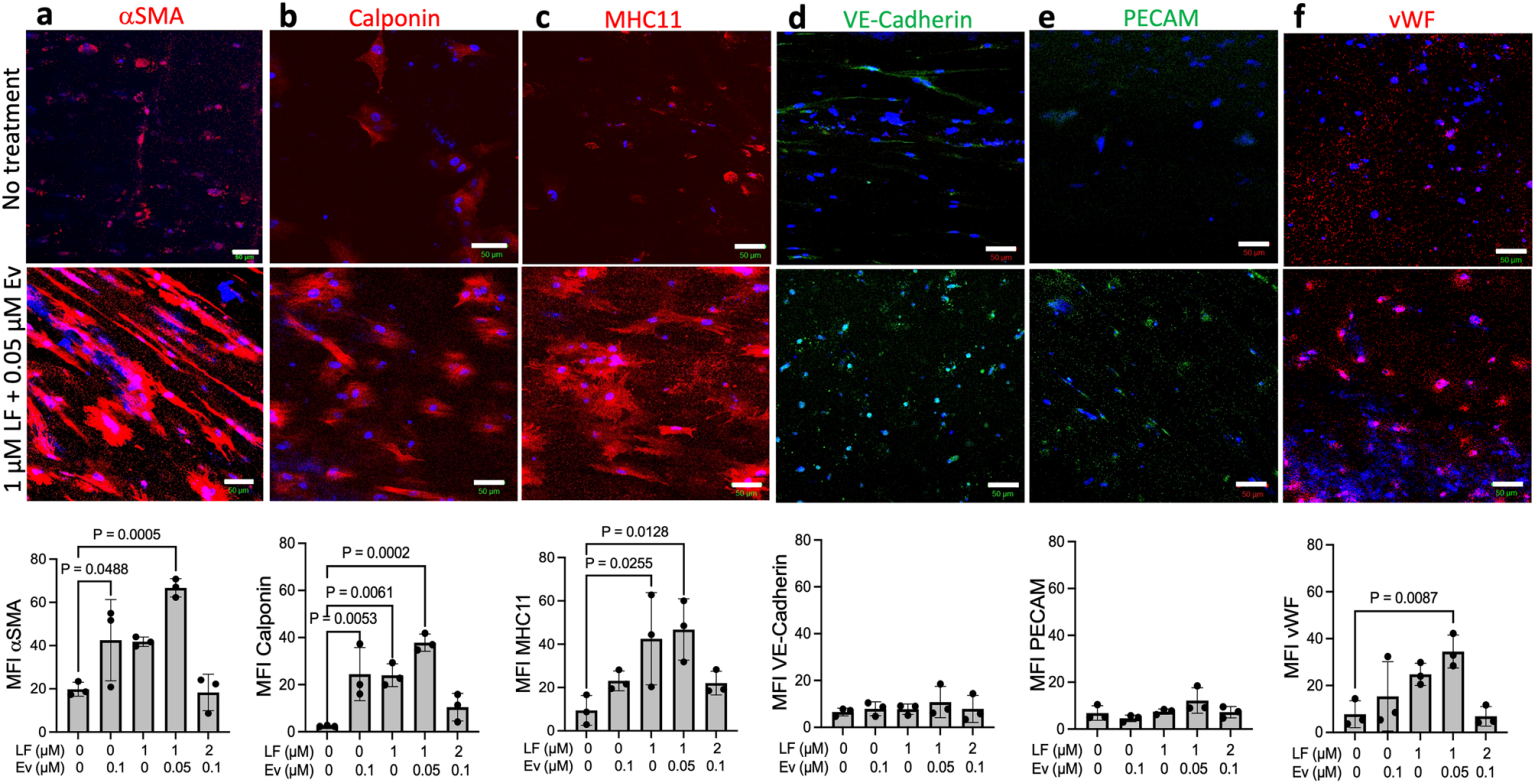
Lonafarnib and Everolimus treatment improves expression of contractile proteins in HGPS TEBVs. HGPS 167 CL2 TEBVs were matured for 3 weeks, treated with Lonafarnib (LF) and Everolimus (Ev) for 1 week and then processed for immunostaining. Representative images and quantification of SMC markers α-smooth muscle actin (αSMA, **a**), calponin (**b**), and myosin heavy chain-11 (MHC11, **c**) and endothelial markers vascular endothelial cadherin (VE-Cadherin, **d**), platelet endothelial cell adhesion molecule (PECAM, **e**), and von Willebrand factor (vWF, **f**). Scale bar: 50 µm. Data presented as mean ± SD. N = 3 experiments per group. Results are compared to the reference condition without Lonafarnib and Everolimus using Dunnett’s test. Representative images for the 1 µM Lonafarnib treatment, 0.1 µM Everolimus treatment, and 2 µM Lonafarnib + 0.1 µM Everolimus treatment conditions are shown in Fig. S11.

We previously showed that HGPS TEBVs express decreased levels of SMC and EC markers compared with healthy TEBVs^10^. Lonafarnib significantly increased the expression of contractile SMC proteins calponin and myosin heavy chain-11 (MHC11) in HGPS TEBVs, while Everolimus significantly increased α-smooth muscle actin (αSMA) and calponin expression (Fig. 5a–c). Treatment with a combination of 1 µM Lonafarnib and 0.05 µM Everolimus combined the benefits of both single-drug treatments, resulting in significantly increased levels of αSMA, calponin, and MHC11 (Fig. 5a–c). Neither Lonafarnib alone nor Everolimus alone significantly improved endothelial cell markers vascular endothelial cadherin (VE-Cadherin), platelet endothelial cell adhesion molecule (PECAM), or von Willebrand factor (vWF) (Fig. 5d–f). While combination treatment with 1 µM Lonafarnib and 0.05 µM Everolimus did not affect VE-Cadherin or PECAM expression (Fig. 5d,e), this treatment did significantly elevate vWF expression (Fig. 5f). Treatment with the toxic combination of 2 µM Lonafarnib and 0.1 µM Everolimus resulted in low expression levels of all 6 proteins which did not differ from untreated HGPS TEBVs (Fig. 5a–f). Representative images of αSMA, calponin, MHC11, VE-Cadherin, PECAM, and vWF expression with Lonafarnib treatment, Everolimus treatment, and the toxic combination are shown in Fig. S11.

### Lonafarnib and Everolimus mitigate disease pathology in HGPS TEBVs

**Figure 6:**
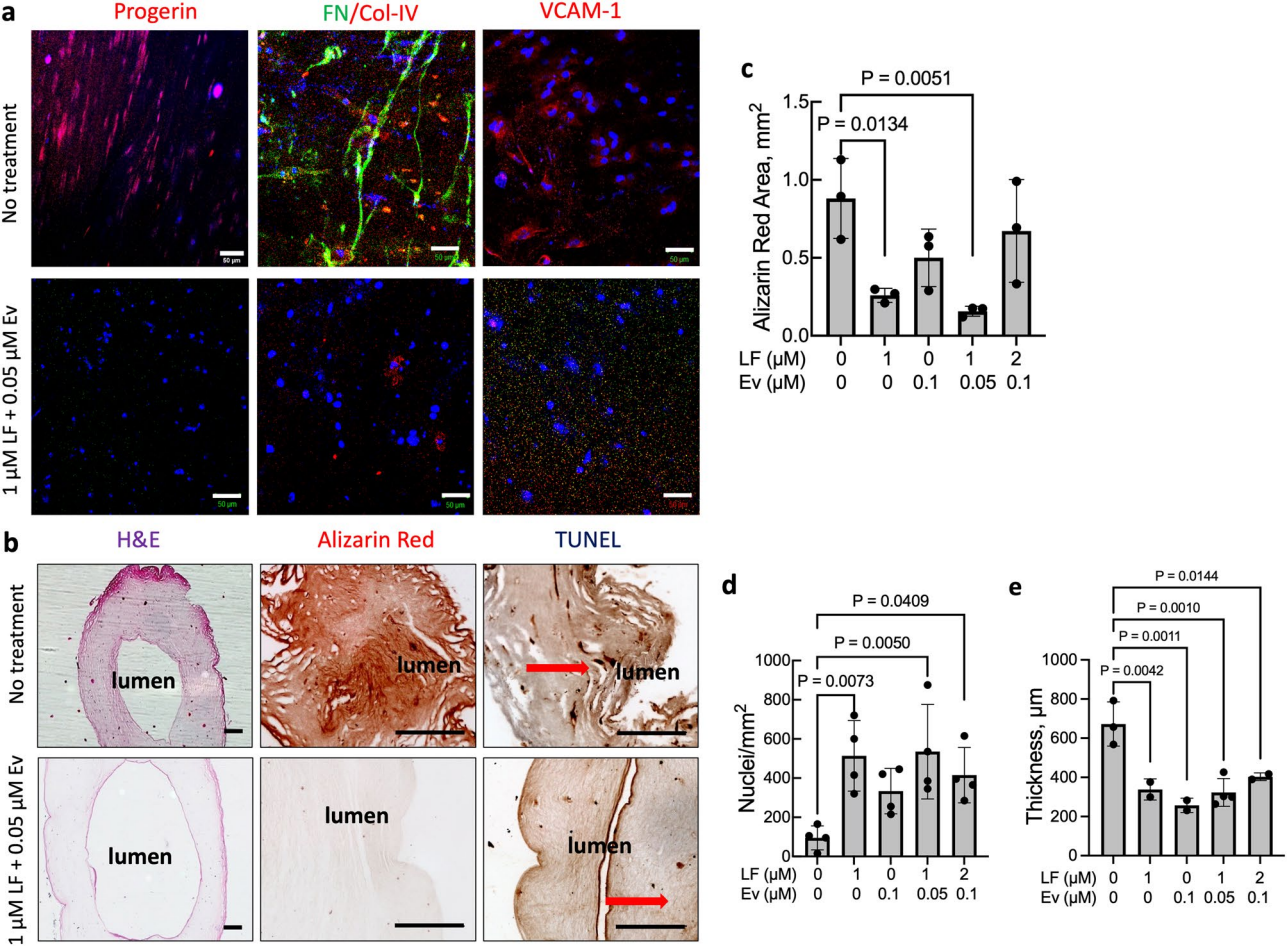
Lonafarnib and Everolimus treatment mitigates HGPS TEBV pathology. Representative immunofluorescence images of (**a**) progerin, fibronectin (FN), collagen IV (Col-IV), vascular cell adhesion molecule-1 (VCAM-1), and (**b**) H&E staining, alizarin red staining, and TUNEL staining in HGPS 167 CL2 TEBVs matured for 3 weeks then treated with 1 µM Lonafarnib (LF) + 0.05 µM Everolimus (Ev) for 1 week. Scale bar 50 µm for progerin, FN/Col-IV, and VCAM-1, and 200 µm for H&E, alizarin red, and TUNEL. (**c**) Quantification of alizarin red-positive area in untreated and treated HGPS 167 Cl2 TEBVs. (**d**) Nuclei density and (**e**) TEBV thickness in untreated and treated HGPS 167 CL2 TEBVs. Data presented as mean ± SD. N = 3 TEBVs per group for alizarin red and 4 TEBVs per group for cell density. *p < 0.05, **p < 0.01 compared with untreated case. Representative images for the 1 µM Lonafarnib treatment, 0.1 µM Everolimus treatment, and 2 µM Lonafarnib + 0.1 µM Everolimus treatment conditions are shown in Figs. S12 and S13.

We assessed the effects of Lonafarnib and Everolimus on disease pathology using the HGPS TEBV model^10,35^. HGPS TEBVs express progerin, stain strongly for extracellular matrix proteins fibronectin and collagen IV, and express the inflammatory marker vascular cell adhesion molecule 1 (VCAM-1) (Fig. 6a). Based on comparison of immunostaining images, treatment with Lonafarnib alone did not affect progerin expression, whereas treatment with Everolimus alone and Everolimus in combination with Lonafarnib reduced progerin expression in HGPS TEBVs (Fig. 6a, Fig. S12a). Additionally, Everolimus alone was insufficient to reduce fibronectin, collagen IV, or VCAM-1 expression, while Lonafarnib alone or in combination with Everolimus reduced expression of all 3 proteins (Fig. 6a, Fig. S12a,b). Treatment with the toxic combination of 2 µM Lonafarnib and 0.1 µM Everolimus did not reduce expression of fibronectin, collagen IV, or VCAM-1 and resembled the untreated HGPS case (Fig. S12a,b).

Compared with healthy TEBVs^35^, HGPS TEBVs displayed lower medial cell density, stained strongly for Alizarin Red, and showed increased TUNEL staining (Fig. 6b–d and Fig. S13). Treatment with Lonafarnib alone reduced calcification as indicated by Alizarin Red staining and increased medial cell density but did not reduce apoptosis marked by TUNEL staining (Fig. 6c,d, Fig. S13b). Treatment with Everolimus alone showed little improvement in calcification (Alizarin Red) or in TUNEL staining (Fig. 6c, Fig. S13b). Treatment with the toxic combination of 2 µM Lonafarnib and 0.1 µM Everolimus did not significantly reduce Alizarin Red staining and resulted in increased TUNEL staining (Fig. 6c, Fig. S13b). Everolimus alone was also not sufficient to significantly increase medial cell density in the TEBVs (Fig. 6d). Treatment with a combination of 1 µM Lonafarnib and 0.05 µM Everolimus significantly decreased Alizarin Red staining, increased cell density, and reduced TUNEL staining (Fig. 6c,d, Fig. S13b). H&E staining indicated that all treatment conditions resulted in reduced wall thickness in HGPS TEBVs (Fig. 6e) and comparable to levels reported for TEBVs made with non-HGPS viECs and viSMCs^10^.

We assessed expression of ATG7, LC3-I/LC3-II and p62 which are all associated with autophagy and are upregulated after Everolimus treatment in progerin-expressing cells to help clear progerin^26^. Immunohistochemical staining was done as described previously^36^ and shows that HGPS TEBVs treated with Everolimus alone or in combination with Lonafarnib at both therapeutic and toxic doses express all three autophagy proteins at more abundant levels compared to untreated controls (Fig. S14). These results are consistent with studies of progerin clearance in 2D fibroblast cultures from HGPS patients^37^. The presence of these autophagy associated proteins along with reduced level of progerin after Everolimus treatment in HGPS TEBVs suggests that Everolimus helps clear progerin through autophagy.

## Discussion

In this study, we showed that a TEBV model of HGPS, together with 2D cell culture, can assess the effect of a combination of drugs on cell and blood vessel function. In HGPS viSMCs and viECs, Everolimus treatment reduced progerin expression and misshapen nuclei in a dose-dependent manner. Everolimus treatment also improved proliferation in one donor and reduced ROS levels and DNA double stranded breaks in both donors. Lonafarnib had a more limited effect on these phenotypes which varied between donors. These results are consistent with studies that showed that rapamycin decreases DNA damage in HGPS fibroblasts while Lonafarnib does not, and that rapamycin also decreases ROS levels in HGPS fibroblasts^38,39^. Treatment with a combination of Lonafarnib and Everolimus showed a beneficial effect upon SMC proteins and vasoactivity.

Progerin expression varies among cell and tissue types and SMCs are among the highest progerin-expressing cells, making them a major target of study in HGPS^13,14^. However, less is known about the role of the endothelium in the disease, despite the well-established role of EC senescence and dysfunction in non-HGPS atherosclerosis^13^. Over 85% of in vitro pre-clinical studies of HGPS therapeutics between 2010 and 2020 were performed with fibroblasts from HGPS patients^40^. HGPS fibroblasts accumulate double stranded DNA breaks over several passages and have elevated ROS levels^41–43^. Further, *NRF2* transcription and nuclear location are affected by progerin expression^44^ and antioxidant treatment of HGPS fibroblasts alleviates characteristic HGPS nuclear abnormalities^45^. HGPS ECs show reduced NO production, impaired mechanosensitivity, and increased inflammation^13,46,47^.

Mice in which only ECs express the LMNA^G609G/G609G^ mutation exhibit cardiac fibrosis^13,46^ and a pro-inflammatory senescence associated secretory phenotype (SASP)^48^. Mice in which only the endothelial cells express progerin exhibit reduced vasodilation^47^ as well as increased collagen and adventitial thickening, which is found in HGPS patients^13^. Further, in the murine model in which only ECs express the LMNA^G609G/G609G^ mutation, the myocardin-related transcription factor A (MRTFA) binds to *NOS3*, which inhibits NO release. Blocking MRFTA restores NO activity^48^. Likewise, in this murine HGPS model, progerin reduces SIRT7 levels in ECs and vascular EC specific SIRT7 overexpression significantly reduced inflammation and promoted survival^47^. Thus, restoring EC function significantly improves vascular function and could improve overall survival of individuals with HGPS^47^. A limitation of these animal studies is that most do not replicate the human vascular pathology.

The HGPS TEBV system models key functional features of arteries under physiological conditions and replicates pathology observed in the disease. As we showed previously^10^, iPSC-derived viSMCs express high levels of αSMA, calponin and MHC11, a terminal marker of SMC differentiation. The viSMCs also exhibit contractile potential that is increased compared with a previous generation of the HGPS TEBV model we reported, resulting in increased vasoconstriction and vasodilation in healthy TEBVs and a larger detectable difference in vasoactivity between healthy and HGPS TEBVs^35^. The viECs express CD31, CD144 in 99% of the cells, as well as VEGF receptor 2 and von Willebrand Factor^10^. In healthy TEBVs, viECs produce nitric oxide which results in TEBV vasodilation. Limitations of the model are that the iPSC-derived cells are not as mature as primary cells and TEBVs do not incorporate elastin. Our experiments only tested the effects of Lonafarnib and Everolimus at a limited timescale of 7 and 14 days, but the TEBV model can show the response to therapeutics.

Despite the large influence of shear stress on EC phenotype, only one other study explored HGPS ECs under shear stress conditions and showed reduced NO production in HGPS ECs compared with healthy cells^49^. We previously showed that HGPS viECs exhibit abnormal nuclear morphology, impaired tube formation, and blunted shear stress-responsive gene expression^10^. We also reported a specific role of the endothelium in HGPS TEBV vasoactivity reduction and inflammatory protein expression^10^. Here we showed that HGPS viECs from two donors exhibit characteristics of endothelial dysfunction including elevated ROS levels, DNA damage, and reduced NO production when exposed to physiological shear stress. These results support the growing body of data that indicate that ECs may play a role in mediating HGPS atherosclerotic development.

Limited results exist on the effect of both Lonafarnib and Everolimus on ECs, SMCs, or the vessel wall. Human coronary ECs exogenously expressing progerin exhibit increased oxidative stress and DNA damage^48^. Treatment of these cells with zoledronate and pravastatin, which respectively block farnesylation and geranylation, reduces the abundance of the pathogenic progerin protein and consequently decreases oxidative stress. Human umbilical vein endothelial cells overexpressing progerin exhibited increased cell loss after 72 h exposure to 12 dynes/cm^2^ which was reversed after addition of Lonafarnib^50^.

In the current study, Lonafarnib and Everolimus treatment rescued NO production under shear stress conditions to healthy levels both when administered alone and in combination. Lonafarnib also significantly increased expression of shear stress-responsive genes to healthy levels or higher while Everolimus had a more limited effect. These results are consistent with a recent study that showed Lonafarnib improves shear stress adaptability in progerin-expressing ECs^50^. In accordance with reduced NO levels after exposure to shear stress, HGPS viECs also exhibited reduced expression of the *NOS3* gene. However, while NO levels increased under shear stress in healthy viECs and treated HGPS viECs, this did not correlate with a significant increase in *NOS3* levels. This indicates that the changes in NO production may be due to causes other than gene regulation such as post-translational modifications of the NOS3 protein (eNOS) which affects its activity*.* HGPS iPSC-derived ECs express lower levels of total eNOS protein as well as increased abundance of phosphorylated threonine 495 eNOS, which inhibits eNOS activity^49^.

HGPS patients develop severe atherosclerosis with vascular dysfunction, stiffening, calcification, inflammation, fibrotic plaque formation, and medial SMC depletion^51^. We previously showed that compared with healthy TEBVs, HGPS TEBVs exhibit reduced vasoactive response to phenylephrine and acetylcholine, decreased contractile markers indicating SMC loss, progerin expression, inflammatory marker expression, and excess ECM deposition^10^. Lonafarnib alone was sufficient to improve endothelium-dependent vasodilation in response to acetylcholine, contractile marker expression, ECM deposition, calcification, inflammatory marker expression, and cell retention. However, Lonafarnib alone did not significantly affect vasoconstriction in response to phenylephrine, progerin expression, or TUNEL expression. In contrast, Everolimus alone was only sufficient to decrease progerin expression, improve vasoconstriction in response to phenylephrine, and increase some contractile markers, but did not affect ECM deposition, inflammatory marker expression, or calcification. These results are consistent with our previous report that Everolimus did not improve calcification or apoptosis in a previous HGPS TEBV model^35^. Treatment with both 1 µM Lonafarnib and 0.05 µM Everolimus combined the beneficial effects of both therapeutics and resulted in a significant increase in endothelial marker vWF and decrease in apoptosis (TUNEL expression), which were not improved by either drug alone. Treatment with the toxic combination of 2 µM Lonafarnib and 0.1 µM Everolimus did not improve disease characteristics and exacerbated TUNEL expression, indicating the importance of proper dosing when combining the two therapies to avoid toxicity. Since the treatment was administered after maturation of the HGPS TEBVs, these results indicate that drug treatment can reverse the phenotypes we tested after the onset of symptoms.

Our results indicate that Everolimus decreases progerin expression, leading to a reduction in ROS levels and DNA damage and improvement in vascular cell proliferation. Everolimus treatment also improves contractile marker expression in HGPS TEBVs and strengthens vasoconstriction. While Everolimus does enhance NO production in HGPS viECs exposed to shear stress, this did not translate to a significant improvement in EC-dependent vasodilation in HGPS TEBVs. The beneficial effects of Everolimus on TEBV structure and function are due, in part, to increased autophagy (Fig. S14). In contrast, Lonafarnib not only improves NO production in HGPS viECs exposed to shear stress, but also significantly increases expression of shear stress-responsive genes. This correlates with a significant improvement in vasodilation in Lonafarnib-treated HGPS TEBVs. Lonafarnib also dominates the positive effect on ECM deposition, cell retention, inflammatory marker expression, and calcification. Lonafarnib was effective at increasing viSMC proliferation.

The Lonafarnib dose in this study (1 µM) is below the typical mean plasma levels of HGPS patients on this drug (1.7–2.8 µM)^15^, but is still effective in improving cell and TEBV function. The dose of Everolimus that led to significant effects in 2D and TEBV studies, 0.05 µM, is slightly less than that used in other in vitro studies^26,29^. For the ongoing clinical trial with Lonafarnib and Everolimus, plasma levels of Everolimus are reported to be comparable to values in other studies with this drug^52^. Reported mean levels in cancer studies^53^ are as high as 6.26–12.52 nM, which is on the lower level of effectiveness in this study. Plasma Everolimus levels between 18 and 27 nM are reported to cause oral mucosal ulcers in cancer patients^54,55^. Everolimus does not appear to cause any vascular problems^56^ and may have some additional beneficial anti-inflammatory effects^57^. Lonafarnib is used to treat infections and side effects include nausea and diarhea^58^. Other than the results of this study, there is no information on adverse effects of the combination of Lonafarnib and Everolimus. While normal TEBVs may be a way to test drug toxicity, the HGPS TEBVs appear to be more sensitive than the healthy TEBVs. Thus, we did not observe toxicity with 0.05 µM EV and 1 µM LF for HGPS TEBVs for this time period.

Overall, our results show that combining Lonafarnib and Everolimus resulted in additional benefits over either therapy alone (Table S4). However, proper dosing of the drugs is essential to identify the therapeutic window. Since calcification would affect vessel stiffness, the most likely vascular response detected would be a decrease in pulse wave velocity. While such changes have been observed with Lonafarnib^15^, detecting an additional beneficial effect of the drug combination may be challenging. Additional study is warranted to better understand the long-term effects of these therapies and how they might interact in different tissues.

## Materials and methods

### Experimental design

We tested phenotypes previously identified in HGPS fibroblasts or mice but not tested in vascular cells or human-derived blood vessels, including reactive oxygen species levels, proliferation, and DNA damage, endothelial shear stress response, blood vessel vasoactivity, progerin expression, extracellular matrix deposition, inflammation, calcification, and apoptosis. To test the effects of Lonafarnib and Everolimus on these phenotypes, we performed studies in iPSC-derived smooth muscle cells (viSMCs), endothelial cells (viECs), and tissue engineered blood vessels. All experiments were independently performed at least 3 times and 2D cell culture experiments were repeated in two HGPS donors.

### Cell culture and isolation

Primary skin fibroblasts with the classic *LMNA* Exon 11 1824 C>T HGPS mutation (167 CL2) and control primary skin fibroblasts (168 CL2) were provided by the Progeria Research Foundation and converted to iPSCs by the Duke iPSC Shared Resource Facility. iPSCs with the classic *LMNA* Exon 11 1824 C>T HGPS mutation (003 CL1D) were provided by the Progeria Research Foundation. The Duke University Health System Institutional Review Board has determined that the protocol using these cells meets the definition of research not involving human subjects as described in 45 CFR 46.102(f), 21 CFR 56.102(e) and 21 CFR 812.3(p) and satisfies the Privacy Rule as described in 45CFR164.514. iPSCs were maintained in feeder-free conditions on hESC-qualified Matrigel (BD biosciences) in mTeSR Plus (StemCell Technologies). Cells were passaged at 80–90% confluency with 0.5 mM EDTA (Invitrogen) for maintenance culture or with Accutase (StemCell Technologies) and 10 µM ROCK inhibitor Y-27632 (Tocris Bioscience) for differentiation.

### viSMC and viEC differentiation

viSMCs and viECs were differentiated using a modified protocol as previously described^10,59^. iPSCs were dissociated on day 0 with Accutase and replated on Matrigel-coated plates at 37,000 cells/cm^2^ for viSMC differentiation or 47,000 cells/cm^2^ for viEC differentiation. On day 1, the medium was replaced with mesoderm induction medium consisting of N2B27 medium (1:1 mix of Neurobasal medium and DMEM/F12 with HEPES supplemented with N2 and B27 minus vitamin A, all Gibco) with 25 ng/mL BMP4 (PeproTech) and 8 µM CHIR99021 (Cayman Chemical). The media was not changed for 3 days to induce a mesoderm state.

For viSMC differentiation, media was changed on day 4 to viSMC induction medium consisting of N2B27 medium, 10 ng/mL PDGF-BB (PeproTech), and 2 ng/mL Activin A (PeproTech). The viSMC induction medium was changed daily. On day 6, cells were dissociated with Accutase and re-plated on collagen-coated plates in viSMC media comprised of N2B27 media supplemented with 2 ng/mL Activin A and 2 µg/mL heparin (Sigma-Aldrich) to induce a contractile SMC phenotype. Media was changed every other day and viSMCs were routinely passaged at 80–90% confluency using Accutase onto collagen-coated plates. viSMCs were used between passages 4–6.

For viEC differentiation, on day 4 the media was changed to viEC induction media consisting of Stempro-34 SFM medium (Thermo Fisher), 200 ng/mL VEGF165 (Genscript), and 2 µM forskolin (Sigma-Aldrich). The viEC induction media was changed daily and conditioned media was collected on days 5, 6, and 7. On day 7, viECs were dissociated with Accutase and MACS separated for CD31+CD144+, CD31−CD144+ and CD31+CD144− cells. viECs were fed with viEC conditioned media consisting of 1:1 fresh Stempro-34 SFM and conditioned medium supplemented with 2 µg/mL heparin until the first passage, then cultured in viEC media consisting of Stempro34-SFM supplemented with 50 ng/mL VEGF and 2 µg/mL heparin.

### viEC magnetic activated cell sorting (MACS)

On day 7 of the viEC differentiation, cells were dissociated with Accutase and neutralized with cold Stempro-34 SFM medium. Cells were spun at 1000 rpm for 5 min and then washed with MACS buffer consisting of PBS with 0.5% BSA and 2 mM EDTA. Cells were resuspended in 8 µL buffer per million cells and co-stained for 15 min on ice with 2 µL per million cells each FCR blocking reagent (Miltenyi Biotec), CD31 microbeads (Miltenyi Biotec), and CD144 microbeads (Miltenyi Biotec) antibody. Cells were rinsed once with MACS buffer then resuspended in 1 mL MACS buffer and ran through an LS column attached to a MACS separator (Miltenyi Biotec) to discard CD31-CD144- cells.

### Cell and TEBV drug treatment

Cells and TEBVs were treated with Lonafarnib, Everolimus, or a combination of the two drugs for a total of 7 days. Drugs were added every 2 days with media change for a total of 3 times throughout the treatment. The control “no treatment” condition cells were cultured for 7 days along with the treated cells with media change every 2 days but without any addition of drug to the media.

### viEC parallel plate flow studies

The effect of physiological shear stresses on healthy and HGPS viECs was evaluated as described previously^10,60^. viECs were seeded at a density of 33,000 cells/cm^2^ onto slide flasks (Nunc) or collagen-coated glass slides and allowed to attach and develop a confluent monolayer in the flasks. Once fully confluent, flasks were incorporated in a parallel plate flow chamber connected to a closed circular flow loop^60^. Steady laminar flow was applied using a pre-conditioning treatment as described previously^60,61^ to maintain cell attachment. Once 12 dynes/cm^2^ was reached, cells were maintained in the flow circuit for 24 h. After 24 h under flow, viECs were removed from the circuit and tested for NO production or shear stress-sensitive gene expression.

### RT-PCR and primers

Total RNA was extracted from normal and HGPS viECs using the RNeasy Mini Kit (Qiagen). RNA was reverse transcribed into cDNA using the iScript cDNA Synthesis Kit (Bio-Rad). RT-PCR was performed in triplicate using the iQ SYBR Green Supermix (Bio-Rad) and the CFX Connect Real-Time PCR Detection System (Bio-Rad). Human *GAPDH* served as an internal control (VHPS-3541, Real-time primers). Primer sequences for human *NOS3, KLF2*, *NRF2*, *NQO1*, *TXNRD1*, *GCLM* and *GCLC* are as follows: ***NOS3-F:*** 5′‐CAT CTT CAG CCC CAA ACG GA‐3′, ***NOS3-R:*** 5′‐AGC GGA TTG TAG CCT GCA AC‐3′; ***KLF2-F:*** 5′‐GCA CGC ACA CAG GTG AGA AG‐3′, ***KLF2-R:*** 5′‐ACC AGT CAC AGT TTG GGA GGG‐3′; ***NRF2-F:*** 5′‐AAA CCA CCC TGA AAG CAC AG‐3′, ***NRF2-R:*** 5′‐AGT GTT CTG GTG ATG CCA CA‐3′; ***NQO1-F:*** 5′‐GGC ATT CTG CAT TTC TGT G‐3′, ***NQO1-R:*** 5′‐GGC GTT TCT TCC ATC CTT C‐3′; ***TXNRD1-F:*** 5′‐CTG CGG GAG AAA AAA GTC G‐3′, ***TXNRD1-R:*** 5′‐CAG GGA TGC CCA AGT AAC G‐3′; ***GCLM-F:*** 5′‐TCA GGG AGT TTC CAG ATG TC‐3′, ***GCLM-R:*** 5′‐CAA TAG GAG GTG AAG CAA TG‐3′; ***GCLC-F:*** 5′‐CAA GAG AAG GGG GAA AGG AC‐3′, ***GCLC-R:*** 5′‐GAC CTC GGG CAG TGT GAA C‐3′.

To account for variability among experiments, a normalization factor was calculated by dividing the global average of all conditions within each group by the average of each experiment^10^. Normalized values were calculated for each experiment by scaling each data point by the normalization factor of the experiment.

### DAF-FM DA NO assay

viECs were maintained in static culture in viEC media with or without therapeutics for 6 days. Media was changed every 2 days. On day 6, media was changed on static samples. Other samples were exposed to 12 dynes/cm^2^ for 24 h. On day 7, static and flow samples were exposed to 1 µM DAF-FM diacetate (Thermo Fisher) in serum free viEC media for 30 min at 37 °C. Cells were rinsed once with PBS and then maintained in fresh media for 15 min to allow for de-esterification of intracellular diacetates. Images were taken on a Zeiss 880 Airyscan inverted confocal microscope at 20 × magnification and analyzed for mean fluorescence intensity using FIJI.

### Quantification of misshapen nuclei

The nuclei per field were quantified from Hoechst-stained nuclei using FIJI. The number of misshapen nuclei in each image were identified as those with abnormal protrusions or shape (Fig. S4a). To correct for differences in cell dispersion, the fraction of misshapen nuclei per total number of nuclei in each frame was determined. Three independent experiments were performed, and three image frames were quantified per experiment. Manual analysis of misshapen nuclei was performed with blinded images to prevent bias.

### TEBV fabrication, treatment, and testing

TEBVs were fabricated as previously described^10^. Briefly, 1.5 × 10^6^ viSMCs were dissociated with Accutase, resuspended in 300 µL of viSMC media, and incorporated in a 2.05 mg/mL rat tail collagen type 1 solution (Corning) in 0.6% acetic acid. Serum-free 10 × DMEM (Sigma-Aldrich) was added at a 1:10 ratio to the collagen solution. The pH of the solution was raised to 8.5 by adding 5 M NaOH, causing gelation. Prior to complete gelation, the solution was placed in a 3 mL syringe mold (BD Biosciences) with the stopper completely removed and a two-way luer-lock stopcock attached to the luer-lock end of the syringe. An 800 μm diameter steel mandrel was inserted in the middle of the solution in the syringe and held in the center with parafilm wrapped over the syringe opening. The solution gelled for 30 min at room temperature prior to compression. After gelation, the vessel construct was transferred onto 0.2 μm nylon filter paper (Whatman) on 10 KimWipes. Plastic compression was applied to the construct by suspension in the filter paper for seven minutes to remove over 90% of the water content. Once compressed, TEBVs were placed in custom perfusion chambers and sutured onto grips at both ends.

After gelation and mounting, TEBVs were endothelialized by resuspending 0.5 × 10^6^ viECs in 0.5 mL viEC media. viECs were perfused through the TEBV lumen using 1 mL luer-lock syringes (BD) connected to the grips of the custom chambers. The TEBVs were evenly endothelialized by rotating the chambers on a rotator at a rate of 10 revolutions per hour for 30 min at 37ºC. Chambers were attached to a flow circuit containing a 25 mL media reservoir connected by tubing. Continuous, steady, laminar flow at 2 mL/min was applied to the TEBVs by attaching the perfusion circuit to a peristaltic pump that applied a physiological shear stress of 6.8 dynes/cm^2^ (Masterflex). The TEBVs were matured for up to 5 weeks in viSMC media and the media was changed 3 times per week.

HGPS TEBVs were matured for 3 weeks without treatment to develop the HGPS phenotype, then treated by adding different combinations of Lonafarnib (Progeria Research Foundation) and Everolimus (StemCell Technologies) in the media. Lonafarnib and Everolimus were replenished with each media change, every 2 days for 1–2 weeks. Vasoactivity was tested weekly to determine function before and after treatment.

TEBV vasoactivity was measured at room temperature in the perfusion circuit with the TEBVs and imaged using a stereoscope (AmScope) while being recorded with ISCapture software. After 30 s of normal perfusion, 1 μM phenylephrine was added to a syringe port (Ibidi) integrated in the flow circuit. Five minutes later, 1 μM acetylcholine was added to the same port. TEBV diameter was measured based on images taken prior to phenylephrine addition, 5 min after phenylephrine addition, and 5 min after acetylcholine addition. The diameter at each time point was determined by averaging 4 random widths along the length of the vessel using ImageJ. Vasoconstriction was calculated as the percent change in diameter from the initial diameter at baseline to the diameter after 5 min of exposure to phenylephrine. Vasodilation was calculated as the percent change in diameter from the constricted state to the diameter after 5 min of exposure to acetylcholine.

### Immunostaining

For 2D viSMC and viEC immunostaining analysis, cells were fixed with 4% paraformaldehyde for 15 min and then washed 3 times with PBS. Cells were permeabilized with 0.1% Triton-X for 5 min at room temperature and then rinsed with PBS 3 times. Cells were blocked in 10% goat serum in PBS for 1 h at room temperature. Cells were then incubated with primary antibody at 1:200 in 10% goat serum overnight at 4ºC. Cells were then washed with PBS 3 times and incubated with secondary antibody at 1:500 in 10% goat serum for 2 h at room temperature. Cells were washed 3 times with DPBS and incubated with Hoechst 33342 at 1:1000 in DPBS for 5 min at room temperature. Samples were then washed 3 times with DPBS and imaged on a Zeiss 880 Airyscan inverted confocal microscope at 20X magnification. Images were analyzed using FIJI.

For 3D TEBV immunostaining analysis, TEBVs were fixed in 10% formalin for 10 min while still attached to the chamber grips. TEBVs were then placed in a well-plate and further fixed in 10% formalin for an additional 50 min. TEBVs were washed with DPBS 3 times and cut *en face* to expose the inner lumen. Sections were permeabilized with 0.1% Triton-X for 10 min and then washed 3 times with DPBS. Samples were blocked in 10% goat serum in PBS for 8 h at room temperature, then incubated with primary antibody (1:100) in 10% goat serum overnight at 4 °C. Samples were washed 3 times with PBS and then incubated with secondary antibody (1:500) in 10% goat serum for 1 h at room temperature. Samples were washed 3 times and incubated with Hoechst 33342 (1:1000) in DPBS for 5 min at room temperature. Samples were washed 3 times with DPBS and mounted onto glass slides with FluorSave (Millipore). TEBVs were imaged using a Zeiss 510 inverted confocal microscope at 20× or 40× magnification and analyzed using Zeiss LSM image browser.

Primary antibodies used were rabbit anti-ki67 (Abcam), rabbit anti-calponin (Abcam), rabbit anti-αSMA (Abcam), rabbit anti-MyHC11 (Abcam), rabbit anti-vWF (Abcam), mouse anti-fibronectin (Abcam), rabbit anti-collagen IV (Abcam), mouse anti-ɣH2A.X (Abcam), mouse anti-CD31 (BD biosciences), mouse anti-VE-cadherin (Santa Cruz), rabbit anti-VCAM-1 (Abcam), mouse anti-E-selectin (Santa Cruz), mouse anti LC3 (Axxora/Enzo), rabbit polyclonal anti-ATG7 (Sant Cruz), rabbit polyclonal anti-p62 (Enzo), mouse anti-lamin A/C (Abcam) and rabbit anti-progerin^26^. Anti-MyHC11, anti-VE-Cadherin, and anti-E-selectin were used at 1:100 dilution. Anti-LC3 was used at 1:20 dilution and anti-lamin A/C was used at 1:1000. Other primary antibodies were used at 1:200 dilution. Secondary antibodies used were Alexa Fluor 594 goat anti-rabbit and Alexa Fluor 488 goat anti-mouse (Thermo Fisher) at 1:500 dilution and Hoechst 33342 (Fischer Scientific) at 1:1000 dilution.

### Immunohistochemistry imaging and analysis

TEBVs were fixed in 10% formalin for 10 min attached to the chamber grips to maintain structural integrity. TEBVs were then placed in a well-plate and fixed in 10% formalin for an additional 50 min for a total of one hour of fixation. TEBVs were maintained in DPBS or 70% ethanol until paraffin embedded. Paraffin embedded cross-sections were then stained with hematoxylin/eosin, Alizarin Red, and TUNEL as previously described^35^. Human bone was used as a positive Alizarin Red control and mouse liver was used as a positive TUNEL control. Images were taken on a Nikon Eclipse TE2000-U microscope at 20× magnification using the NIS Elements software and analyzed using ImageJ.

### Live/dead staining

viSMCs and viECs were incubated in their respective media containing 2 μM Calcein AM and 1 μM Ethidium homodimer-1 (Life Technologies) for 1 h at room temperature. viSMCs were then imaged on a fluorescent microscope at 10× and analyzed using ImageJ. Live cells were quantified by determining the maxima of the FITC (green) channel and dead cell counts were quantified by determining the maxima of the TXRED (red) channel. The percent of live and dead cells was determined by dividing the live or dead cell count by the total number of live and dead cells.

### Western blotting

Whole cell lysates were prepared by dissolving cells in Laemmli Sample Buffer containing 5% of 2-mercaptoethanol (Bio-Rad). Antibodies used for immunoblotting include: anti-progerin^26^ with 1:500 dilution, and mouse anti-GAPDH (sc-47724; Santa Cruz) with 1:3000 dilution. Protein samples were loaded on 12% polyacrylamide gels (Bio-Rad) and run at 100 V until the dye front reached the bottom of the gel. The proteins were transferred onto 0.45 μm pore-size nitrocellulose membranes (Bio-Rad) using Turboblot (Bio-Rad). Then, blots were probed with primary and secondary antibodies, and images were developed using ECL (Bio-Rad). Image Lab software (Bio-Rad) was used for image capture and analysis. For each experiment, all conditions (control, DMSO vehicle and Everolimus does) run on the same gel. For each gel, values are normalized to control and then the results of the different gels averaged. Complete gels are shown in Fig. S2.

### DCFDA assay

viSMCs and viECs were cultured with or without therapeutics for 7 days, then washed once with 1X buffer (Abcam). Cells were incubated with 10 µM DCFDA solution (Abcam) diluted in 1× buffer for 5 min at room temperature. After staining, cells were washed with 1× buffer twice and then imaged on a Zeiss 880 Airyscan confocal microscope at 20× magnification. Images were analyzed in FIJI for mean fluorescence intensity.

### Quantification of DCFDA, Ki67, and γH2A.X

For image analysis in FIJI, a threshold was set to identify positive staining for each marker analyzed (DCFDA, Ki67, γH2A.X) and kept standard across all the images. For DCFDA, mean fluorescence intensity was calculated and normalized by the healthy condition to report fold change. For Ki67, positive cells were automatically counted in FIJI based on the positive threshold and divided by the total number of cells to report percent Ki67-positive cells. For γH2A.X, positive foci were automatically counted using FIJI’s particle counting function based on the positive threshold and size and divided by the total number of nuclei to report γH2A.X foci per cell. To report percent γH2A.X-positive nuclei, the number of nuclei with foci was manually counted based on the automatic detection of foci by FIJI overlayed with the Hoechst staining, then this number was divided by the total number of nuclei.

### Statistical analysis

Statistical analysis was performed using JMP Pro 14 (SAS) and GraphPad Prism. Data were analyzed using a one- or two-way ANOVA and post-hoc Tukey test to compare means across groups or Dunnett’s test when comparing means to one control. Repeated measures ANOVA was used for time-dependent assays. Data are represented as mean ± SD with N = number of independent experiments; p ≤ 0.05 was considered significant.

## Supplementary Information

Supplementary Information.

## Data Availability

All data generated or analyzed during this study are included in this published article (and its Supplementary Information files).
