# Supplementary material for: Lonafarnib and everolimus reduce pathology in iPSC-derived tissue engineered blood vessel model of Hutchinson-Gilford Progeria Syndrome
Source: Sci Rep. 2023 Mar 28;13:5032. doi: 10.1038/s41598-023-32035-3 (PMC10050176; doi:10.1038/s41598-023-32035-3)
Supplement: Supplementary file 1 — Supplementary Information. [file 41598_2023_32035_MOESM1_ESM.pdf]

Supplementary Information for
Lonafarnib and Everolimus reduce pathology in iPSC-derived tissue engineered blood vessel model of Hutchinson-Gilford Progeria Syndrome

Nadia O. Abutaleb, Leigh Atchison, Leandro Choi, Akhil Bedapudi, Kevin Shores, Yantenew Gete, Kan Cao, George A. Truskey

Corresponding author George A. Truskey
Email: gtruskey@duke.edu

This file includes:

Figs. S1 to S14
Tables S1 to S4

Figure S1. Cell viability with Lonafarnib and Everolimus treatment.

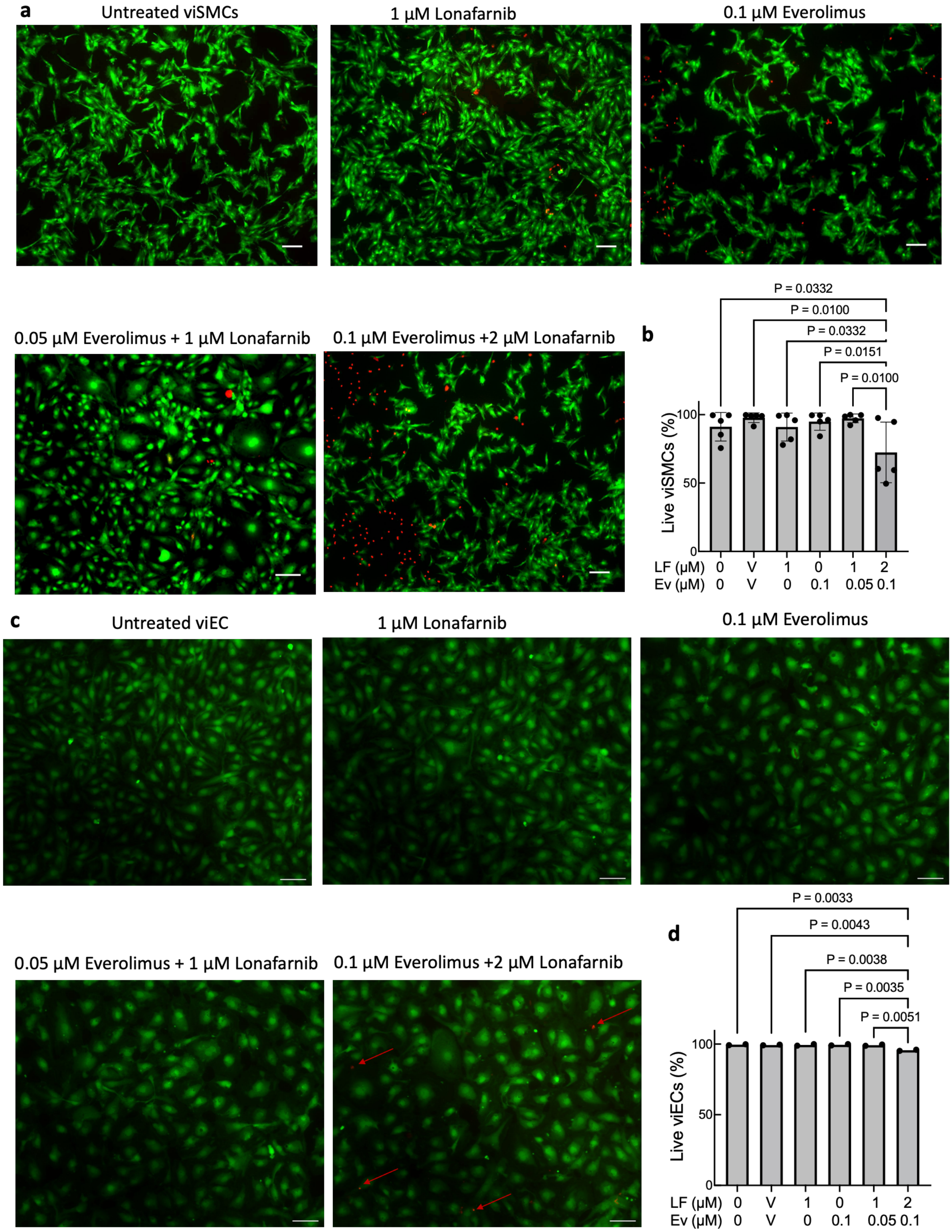

Representative images (**a**, **c**) and quantification (**b**, **d**) of calcein-AM/EthD-1 staining on HGPS 167 CL2 viSMCs (**a**, **b**) and viECs (**c**, **d**) treated with Lonafarnib (LF) and Everolimus (Ev). 'V' indicates DMSO vehicle control. Scale bar 200 μm in **a** and 100 μm in **c**. Arrows in **c** denote EthD-1 to identify dead cells. Mean ± SD, N = 5 per group for viSMCs and N=2 for viECs. Data analyzed by ANOVA followed by post hoc Tukey text.

Figure S2. Entire Western Blots of Progerin Levels for Different Everolimus Concentrations

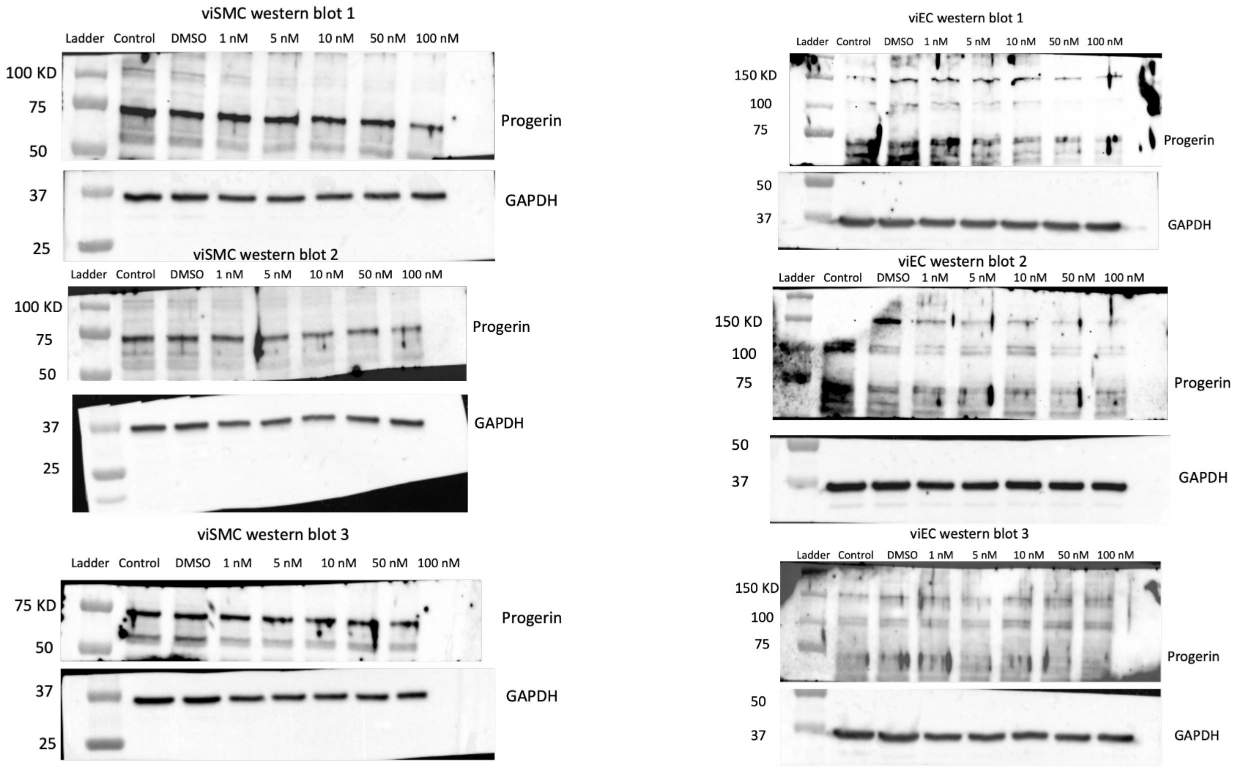

Images of the entire western blots for quantification of progerin levels in Figure 1. Each blot was cut and regions noted separately stained for GAPDH and progerin.

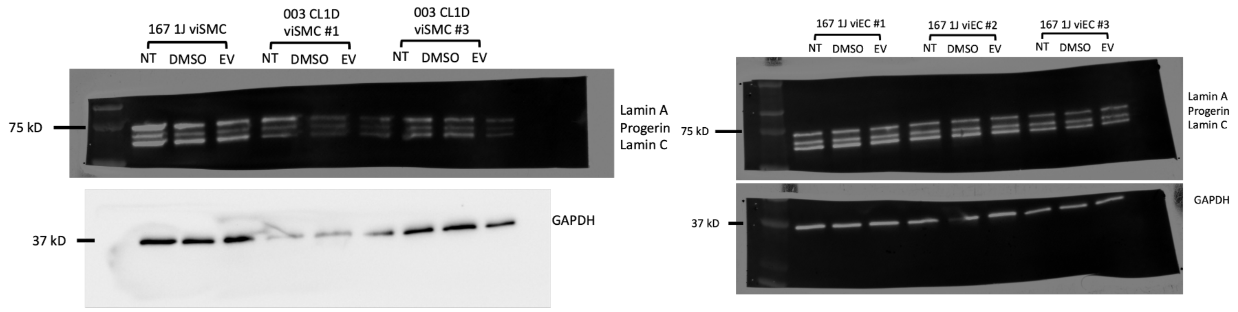

Images of the Western blots for quantification of protein levels in Figure S3. Each blot was cut and regions noted separately stained for GAPDH, progerin, lamin A, and lamin C.

Figure S3. Effect of Everolimus on levels of progerin, lamin A and lamin C in HGPS viECs and viSMCs.

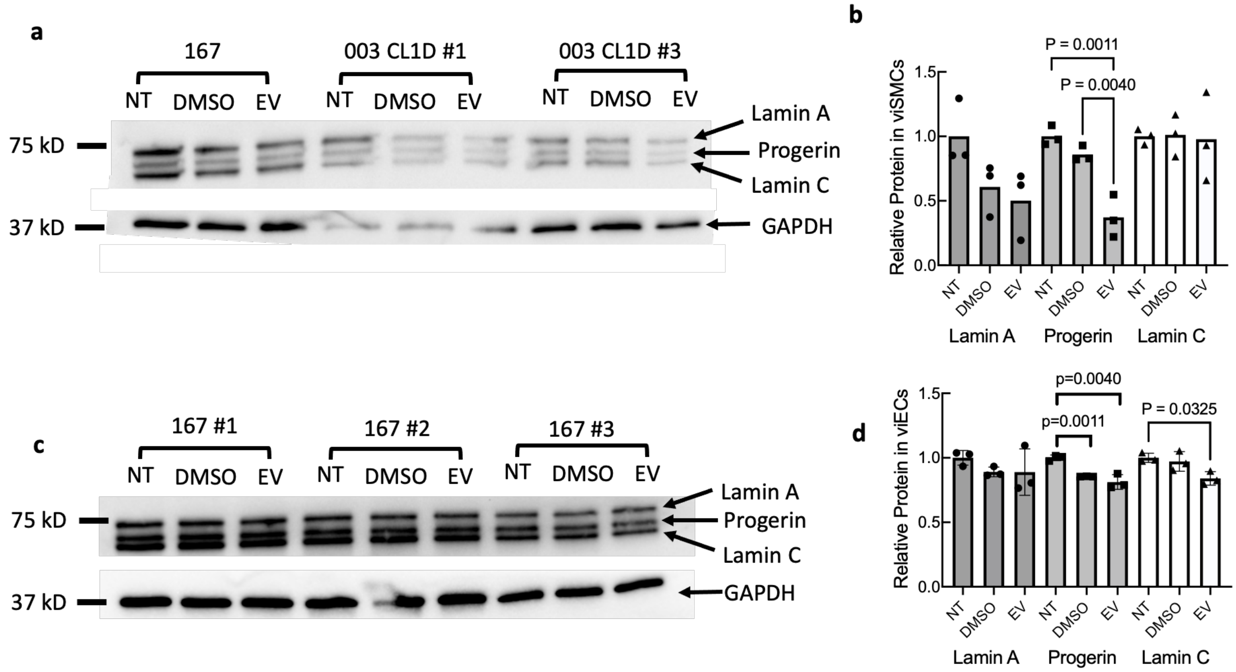

The viSMCs and viECs were incubated for 7 days in media containing 0.1 μM Everolimus as in Figure 1. Western blot was performed using an antibody to lamin A/C for viSMC (**a**) and viECs (**b**) which were quantified by densitometry (**b**, **d**). Data analyzed by ANOVA followed by post hoc Tukey text.

Figure S4. Misshapen HGPS nuclei

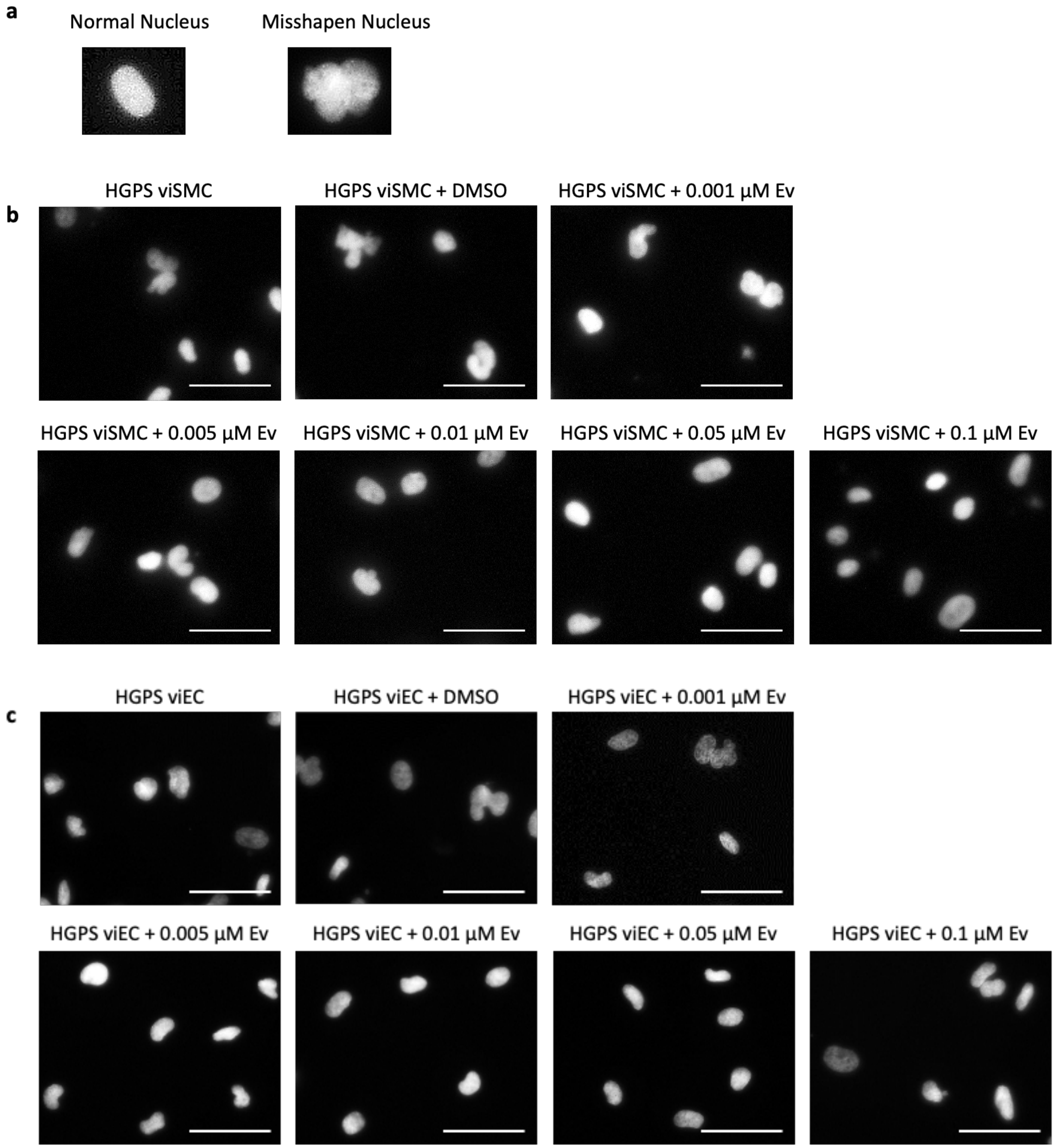

(**a**) Images of normal and misshapen Hoechst-stained HGPS nuclei. Images of Hoechst-stained HGPS viSMC (**b**) and viEC (**c**) nuclei after exposure to the indicated concentrations of Everolimus (Ev) for 7 days, scale bar = 50 µm. Images were sharpened in ImageJ to enhance contrast.

Figure S5. Everolimus reduces ROS levels, increases proliferation, and decreases DNA damage in HGPS 003 CL1D viSMCs and viECs.

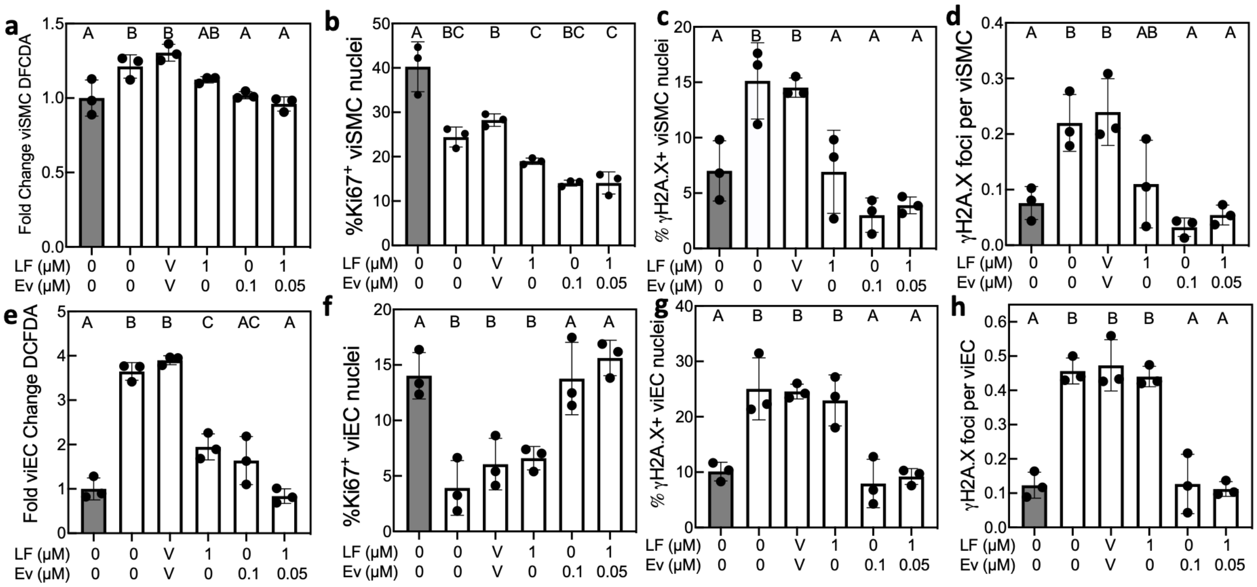

Healthy viSMCs (a-d) and viECs (e-h) and HGPS 003 CL1D viSMCs and viECs treated with different combinations of Lonafarnib (LF) and Everolimus (Ev) for: fold change of DCFDA mean fluorescence intensity compared with healthy in viSMCs (a) and viECs (e); percent positive Ki67 nuclei in viSMCs (b) and viECs (f); percent nuclei with ɣH2A.X foci in viSMCs (c) and viECs (g); and number of ɣH2A.X foci per total cells in viSMCs (d) and viECs (h). V' indicates DMSO vehicle control. Healthy bars are shown in gray and HGPS bars are shown in white. Data presented as mean ± SD. N = 3 experiments per group. Data were analyzed by ANOVA followed by a post hoc Tukey test. Groups connected by the same letter are not significantly different from each other (p>0.05). Groups labeled with different letters are significantly different from each other (p<0.05). Exact p-values for significant differences are provided in Table S3.

Figure S6. Representative images of HGPS viECs and viSMCs treated with DCFDA to detect reactive oxygen species

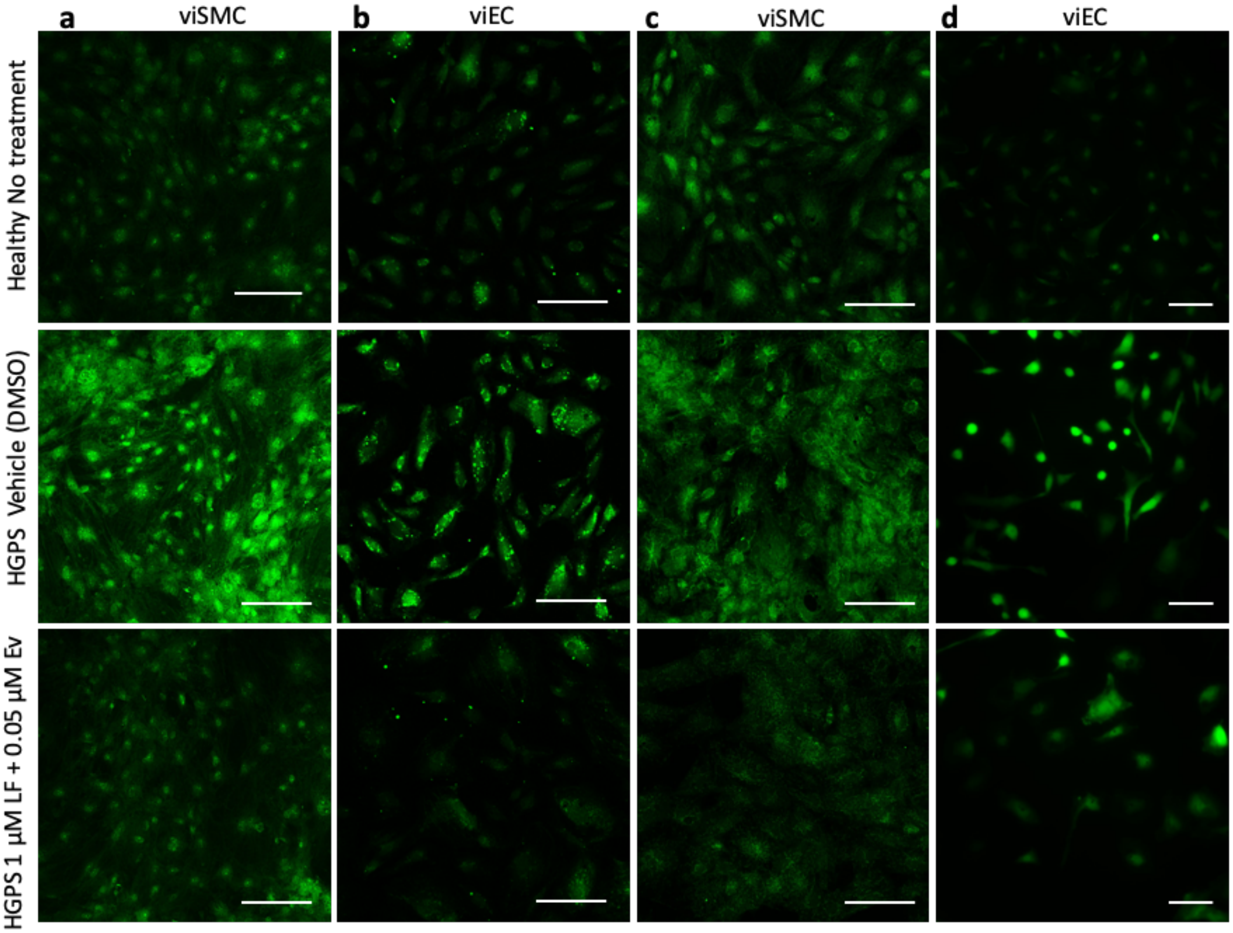

Representative DCFDA images for healthy, HGPS 167 (**a**, **b**), and HGPS 003 (**c**, **d**) viSMCs and viECs with and without LF+Ev treatment. Scale bar 100 µm. Images shown in panels **a, b, c**, and **d** correspond to quantification shown in Figs. 2a, 2e, S5a, and S5e, respectively.

Figure S7. Images of HGPS viEC and viSMC Proliferation as measured using Ki67

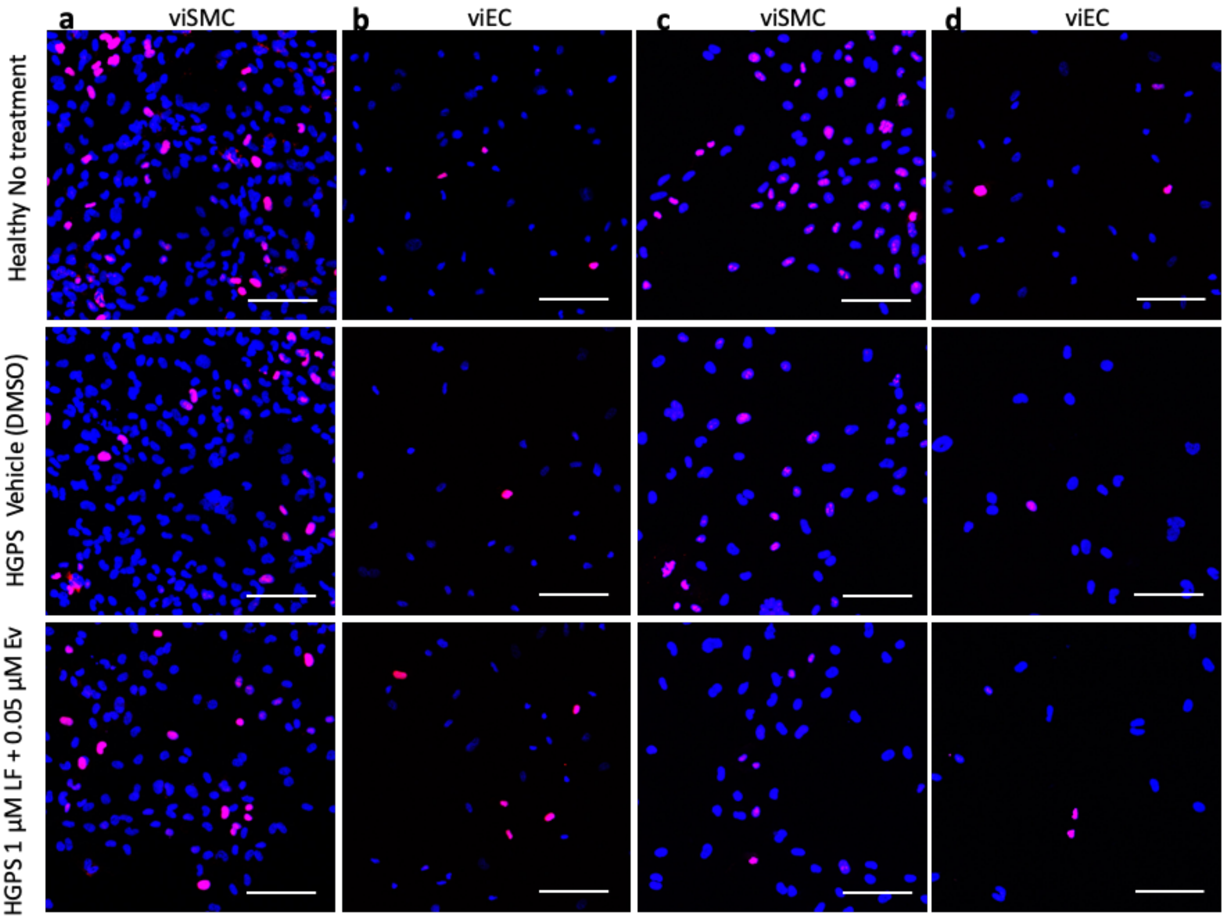

Representative Ki67 images for healthy, HGPS 167 (**a**, **b**), and HGPS 003 (**c**, **d**) viSMCs and viECs with and without LF+Ev treatment. Scale bar 100 µm. Images shown in panels **a**, **b**, **c,** and **d** correspond to quantification shown in Figs. 2b, 2f, S5b, and S5f, respectively. Red staining indicates nuclei staining positively for Ki67.

Figure S8. Images of DNA double-stranded breaks in HGPS viEC and viSMC

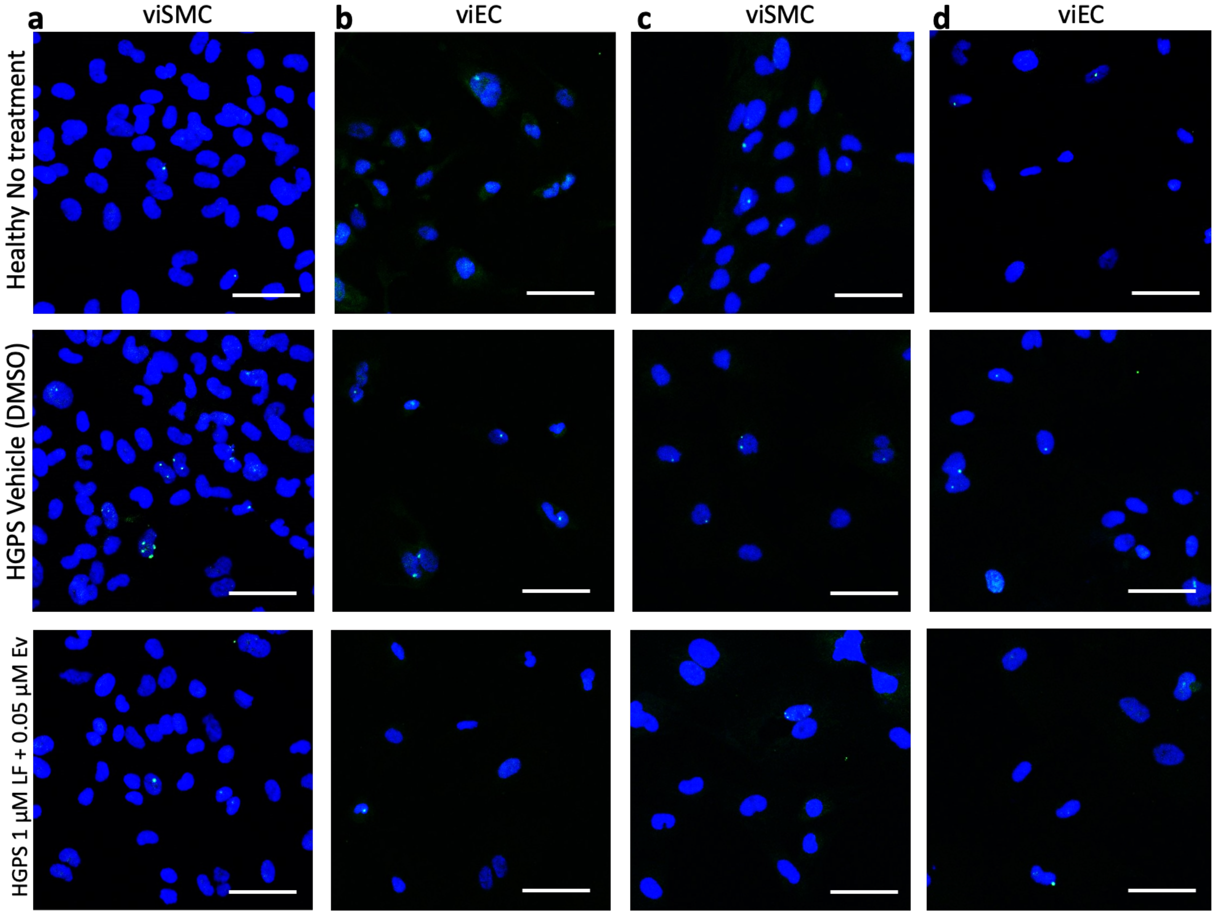

Representative γH2A.X images for healthy, HGPS 167 (**a**, **b**), and HGPS 003 (**c**, **d**) viSMCs and viECs with and without LF+Ev treatment. Scale bar 50 µm. Images shown in panel **a** correspond to quantification shown in Fig. 2c, d, panel **b** to quantification shown in Fig. 2g, h, panel **c** to quantification shown in Fig. S5c, d, and panel **d** to quantification shown in Fig. S5g, h. Green foci indicate positive staining for γH2A.

Figure S9. Images of DAF-FM staining in HGPS viECs after exposure to flow

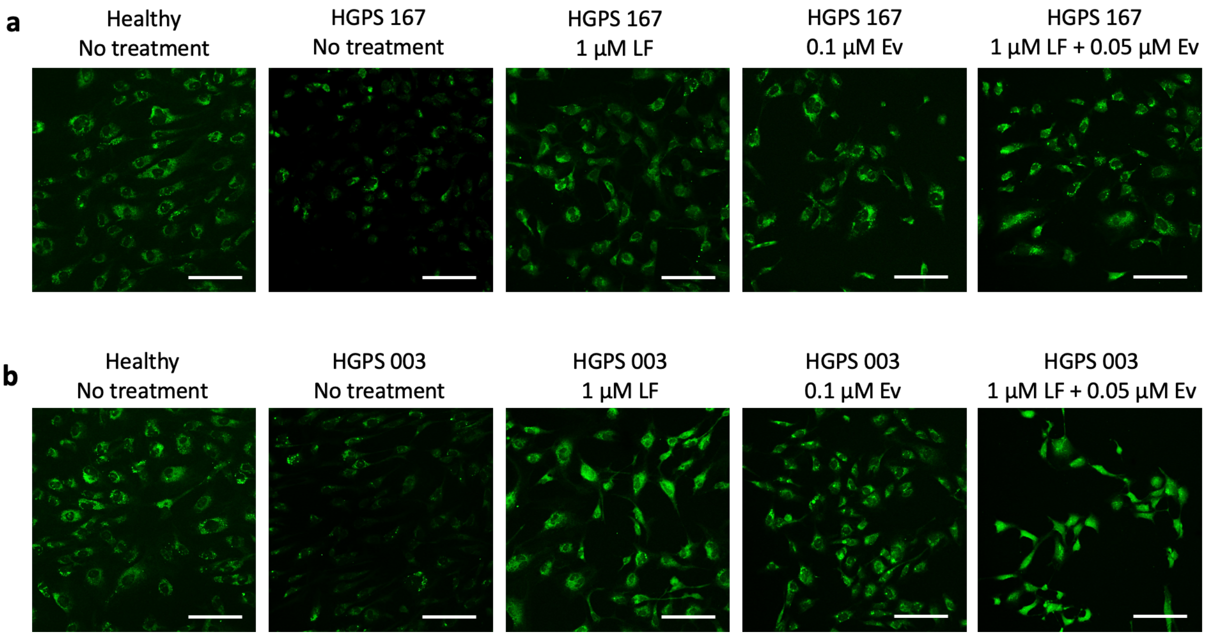

Representative images of DAF-FM diacetate in HGPS 167 (**a**) and HGPS 003 (**b**) viECs exposed to 12 dynes/cm^2 shear stress for 24 hours, with and without 7-day LF and Ev treatment. Scale bar 100 μm. Quantification is shown in Fig. 3**a**, **b**.

Figure S10. 2-week treatment of HGPS 167 CL2 TEBVs with Lonafarnib and Everolimus.

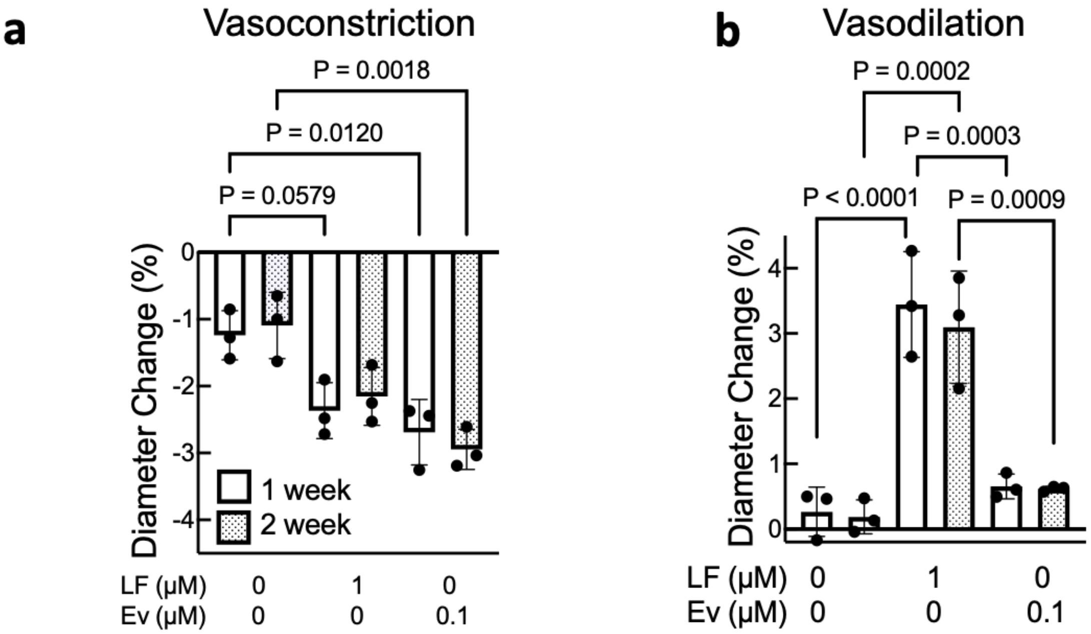

Diameter change in response to 5-minute exposure to (**a**) 1 μM phenylephrine or (**b**) 1 μM acetylcholine of HGPS 167 CL2 TEBVs matured for 3 weeks then treated for 1 or 2 weeks with 1 μM Lonafarnib or 0.1 μM Everolimus. Data presented as mean ± SD. N = 3 TEBVs per treatment. Data analyzed by ANOVA followed by post hoc Tukey text.

Fig. S11. TEBV contractile and endothelial protein expression with Lonafarnib, Everolimus, and toxic combination treatment.

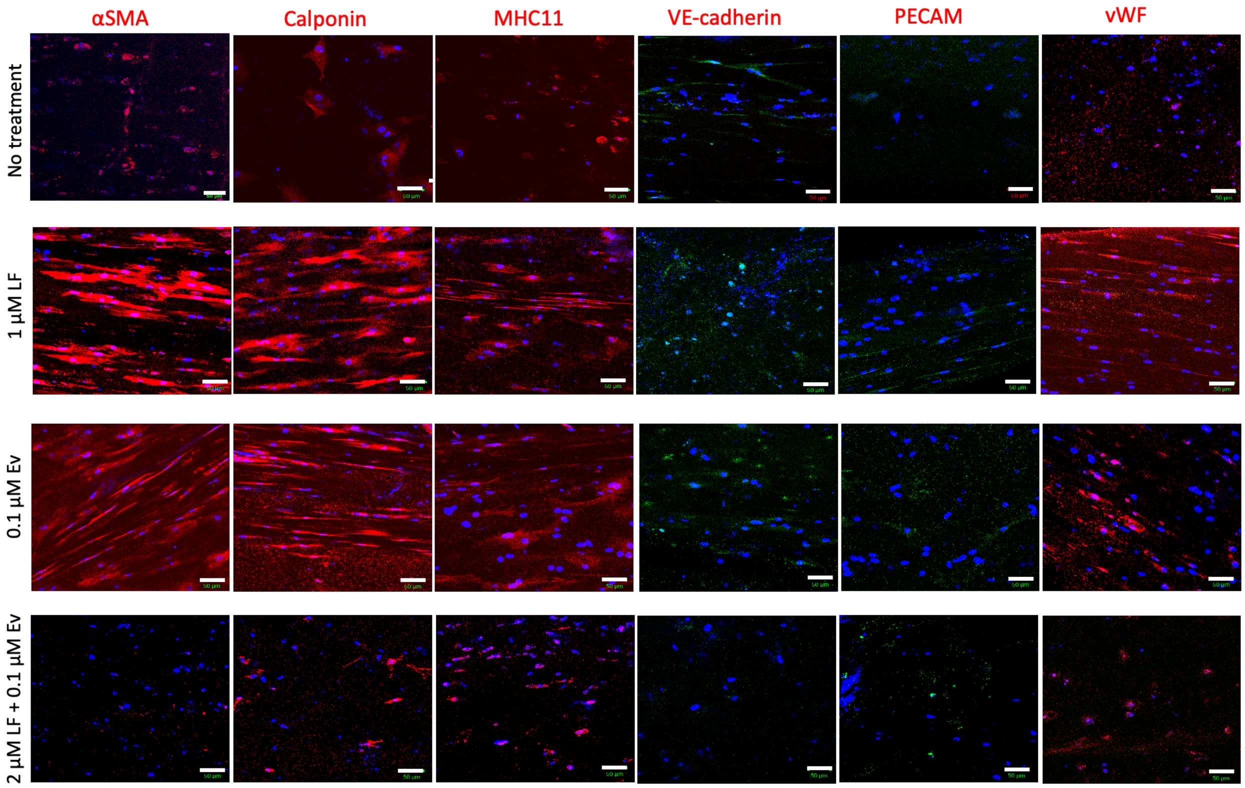

Representative images of SMC markers α-smooth muscle actin (αSMA), calponin, and myosin heavy chain 11 (MHC11) and endothelial markers vascular endothelial cadherin (VE-cadherin), platelet endothelial cell adhesion molecule (PECAM), and von Willebrand factor (vWF) expression in TEBVs matured for 3 weeks then treated with Lonafarnib (LF), Everolimus (Ev), or both for 1 week. Scale bar 50 µm. Representative images for HGPS TEBVs with no treatment shown in Figure 5 are provided again for comparison.

Figure S12. HGPS TEBV Progerin, Fibronectin, Collagen IV and VCAM-1 with Lonafarnib, Everolimus, and toxic combination treatment.

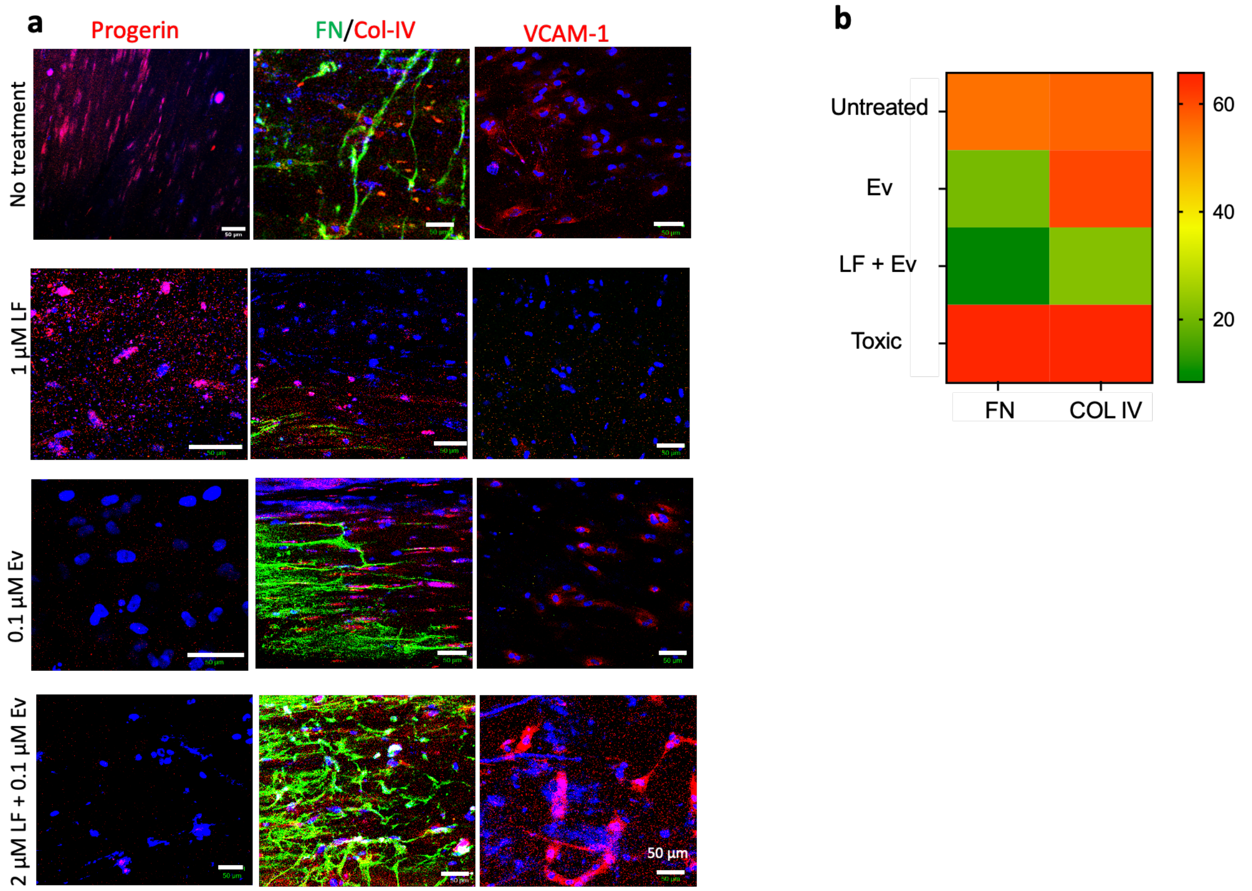

a. Representative images of progerin, fibronectin (FN), collagen IV (Col-IV), and vascular cell adhesion molecule-1 (VCAM-1) expression in TEBVs matured for 3 weeks then treated with LF, Ev, or both for 1 week. Scale bar 50 µm for progerin, FN/Col-IV, and VCAM-1. Representative images for HGPS TEBVs with no treatment shown in Figure 6 are provided again for comparison. **b**. Heatmap showing relative change in FN and COL IV after treatments from staining in a single experiment for each condition.

Figure S13. HGPS TEBV disease pathology with Lonafarnib, Everolimus, and toxic combination treatment.

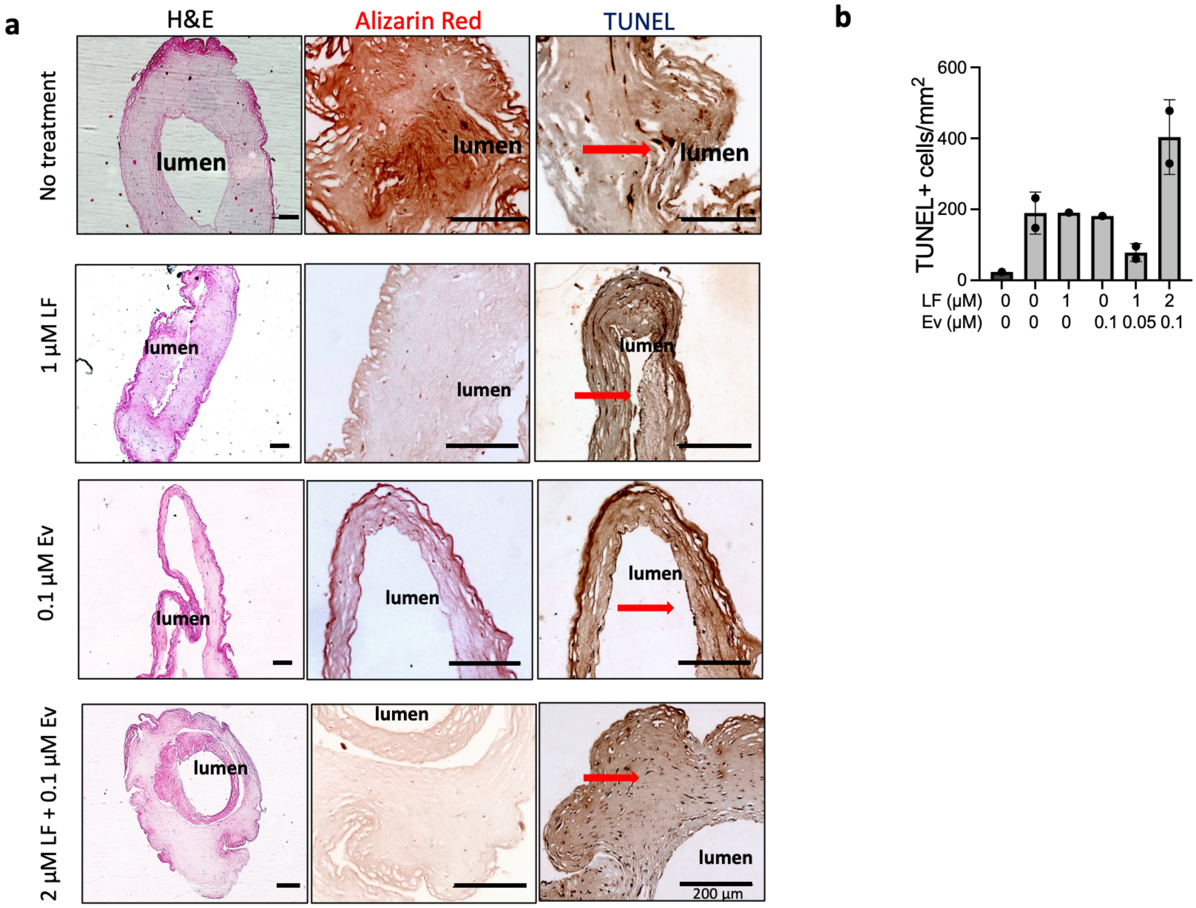

a. Representative images of H&E staining, Alizarin Red staining, and TUNEL staining in TEBVs matured for 3 weeks then treated with LF, Ev, or both for 1 week. Scale bar 200 µm for H&E, Alizarin Red, and TUNEL. Representative images for HGPS TEBVs with no treatment shown in Figure 6 are provided again for comparison. **b**. Trends in TUNEL positive cells treatments. Dark gray bar indicates healthy TEBVs.

Figure S14. Effects of Everolimus on autophagy in HGPS TEBVs

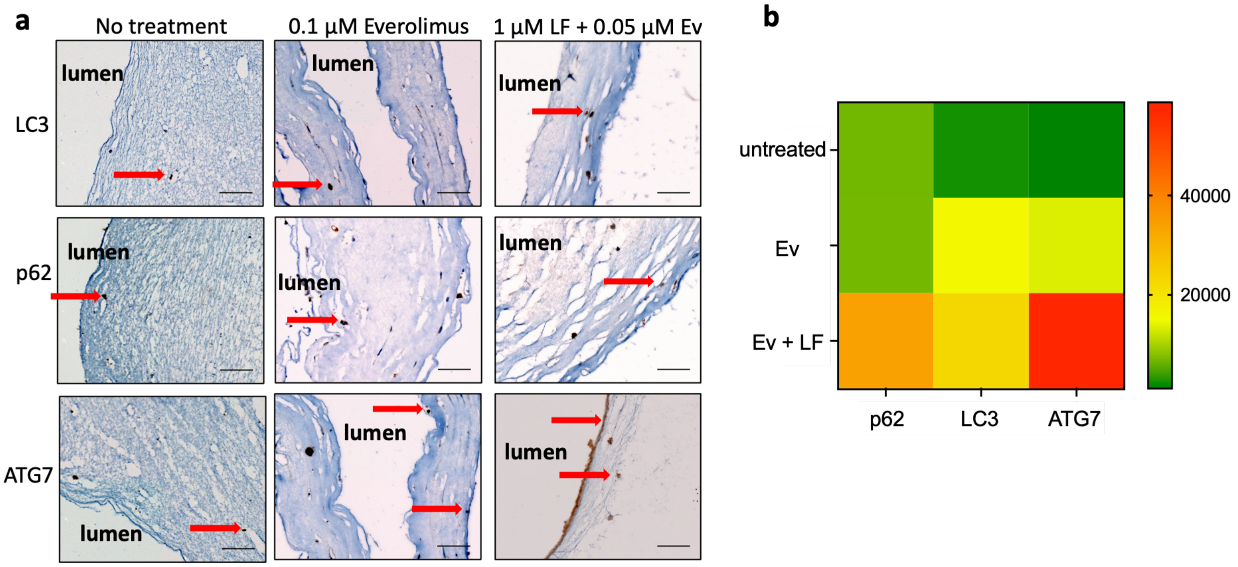

a. Representative images of immunohistological staining in HGPS TEBVs for autophagy proteins LC3-I/LC3-II, p62 and ATG7 after 3 weeks of normal perfusion and 1 week of either no treatment, 0.1μM Everolimus, or 0.05 μM Everolimus and 1 μM Lonafarnib treatment (scale bar, 50 μm). Red arrows indicate LC3, p62 and ATG7-positive cells. **b**. Heatmap showing relative change in autophagy markers after various treatments from a single experiment for each condition.

Table S1. Significant p-values from experiments in Figure 2.

Comparison		Figure Panel							
		2A	2B	2C	2D	2E	2F	2G	2H
Healthy	HGPS NT	0.0022	0.0018	<0.0001	0.0014	<0.0001	0.0117	0.0019	0.0023
Healthy	HGPS DMSO	0.0019	0.0038	<0.0001	0.0061	<0.0001	0.0198	0.0059	0.0096
Healthy	HGPS LF	NS	0.0093	0.0084	0.0199	0.0001	NS	0.0045	0.0156
Healthy	HGPS Ev	NS	NS	NS	NS	NS	NS	NS	NS
Healthy	HGPS LF+Ev	NS	NS	NS	NS	NS	NS	NS	NS
HGPS NT	HGPS DMSO	NS	NS	NS	NS	NS	NS	NS	NS
HGPS NT	HGPS LF	NS	NS	NS	NS	NS	0.0355	NS	NS
HGPS NT	HGPS Ev	0.0187	0.0094	0.0023	NS	<0.0001	0.0003	0.0014	0.0020
HGPS NT	HGPS LF+Ev	0.0260	0.0043	0.0024	NS	<0.0001	0.0047	0.0072	0.0064
HGPS DMSO	HGPS LF	NS	NS	NS	NS	NS	NS	NS	NS
HGPS DMSO	HGPS Ev	0.0158	0.0207	0.0035	NS	<0.0001	0.0005	0.0042	0.0084
HGPS DMSO	HGPS LF+Ev	0.022	0.0092	0.0037	NS	<0.0001	0.0079	0.0228	0.0285
HGPS LF	HGPS Ev	NS	NS	NS	NS	<0.0001	NS	0.0032	0.0136
HGPS LF	HGPS LF+Ev	NS	0.0228	NS	NS	<0.0001	NS	0.0173	0.0463
HGPS Ev	HGPS LF+Ev	NS	NS	NS	NS	NS	NS	NS	NS

Table S2. Significant p-values from experiments in Figure 3.

		Figure Panel							
**Comparison**		3A	3B	3D	3E	3F	3G	3H	3I
Healthy	HGPS NT	0.0015	0.0022	0.0513	0.0013	<0.0001	<0.0001	0.0001	0.0004
Healthy	HGPS DMSO	N/A	N/A	N/A	N/A	N/A	N/A	N/A	N/A
Healthy	HGPS LF	NS	NS	<0.0001	NS	<0.0001	NS	0.0230	0.0245
Healthy	HGPS Ev	NS	NS	<0.0001	0.0007	<0.0001	0.0177	0.0220	0.0016
Healthy	HGPS LF+Ev	NS	NS	<0.0001	0.0067	<0.0001	0.0033	0.0462	0.0126
HGPS NT	HGPS DMSO	N/A	N/A	N/A	N/A	N/A	N/A	N/A	N/A
HGPS NT	HGPS LF	0.0015	0.0017	<0.0001	0.0132	<0.0001	<0.0001	<0.0001	<0.0001
HGPS NT	HGPS Ev	0.0016	0.0002	<0.0001	NS	<0.0001	0.0096	0.0189	NS
HGPS NT	HGPS LF+Ev	0.0074	0.0139	<0.0001	NS	<0.0001	NS	<0.0001	NS
HGPS DMSO	HGPS LF	N/A	N/A	N/A	N/A	N/A	N/A	N/A	N/A
HGPS DMSO	HGPS Ev	N/A	N/A	N/A	N/A	N/A	N/A	N/A	N/A
HGPS DMSO	HGPS LF+Ev	N/A	N/A	N/A	N/A	N/A	N/A	N/A	N/A
HGPS LF	HGPS Ev	NS	NS	<0.0001	0.0061	<0.0001	0.0008	0.0001	<0.0001
HGPS LF	HGPS LF+Ev	NS	NS	<0.0001	NS	0.0267	0.0002	NS	<0.0001
HGPS Ev	HGPS LF+Ev	NS	NS	<0.0001	NS	0.0003	NS	0.0002	NS

Table S3. Significant p-values from experiments in Figure S5.

		Figure Panel							
Comparison		S4A	S4B	S4C	S4D	S4E	S4F	S4G	S4H
Healthy	HGPS NT	0.0215	0.0007	0.0169	0.0297	<0.0001	0.0014	0.0030	<0.0001
Healthy	HGPS DMSO	0.0014	0.0067	0.0280	0.0128	<0.0001	0.0094	0.0038	<0.0001
Healthy	HGPS LF	NS	<0.0001	NS	NS	0.0185	0.0155	0.0094	0.0001
Healthy	HGPS Ev	NS	0.0002	NS	NS	NS	NS	NS	NS
Healthy	HGPS LF+Ev	NS	<0.0001	NS	NS	NS	NS	NS	NS
HGPS NT	HGPS DMSO	NS	NS	NS	NS	NS	NS	NS	NS
HGPS NT	HGPS LF	NS	NS	0.0158	NS	0.0001	NS	NS	NS
HGPS NT	HGPS Ev	0.0387	NS	0.0007	0.0047	<0.0001	0.0018	0.0009	<0.0001
HGPS NT	HGPS LF+Ev	0.0065	NS	0.0014	0.0119	<0.0001	0.0004	0.0018	<0.0001
HGPS DMSO	HGPS LF	NS	0.0386	0.0261	NS	<0.0001	NS	NS	NS
HGPS DMSO	HGPS Ev	0.0024	NS	0.0011	0.0021	<0.0001	0.0019	0.0012	<0.0001
HGPS DMSO	HGPS LF+Ev	0.0005	0.0182	0.0022	0.0052	<0.0001	0.0022	0.0024	<0.0001
HGPS LF	HGPS Ev	NS	NS	NS	NS	NS	0.0195	0.0029	0.0001
HGPS LF	HGPS LF+Ev	NS	NS	NS	NS	0.0059	0.0036	0.0057	<0.0001
HGPS Ev	HGPS LF+Ev	NS	NS	NS	NS	NS	NS	NS	NS

Table S4. Comparison of Lonafarnib and Everolimus treatments alone and in combination. "↑" indicates an increase and "↓" indicates a decrease by the treatment. Within the same row, additional arrows indicate a stronger effect between different treatments.

Characteristic	Lonafarnib	Everolimus	Combination
viECs and viSMCs			
ROS Levels	↓	↓↓	↓↓
Proliferation	none	↑	↑
DNA damage	none	↓	↓
Shear stress-sensitive gene expression	↑↑	↑	↑
eNOS expression under shear stress	none	↑	↑
NO production under shear stress	↑	↑	↑
TEBVs			
Vasoconstriction	none	↑	↑
Vasodilation	↑	none	↑
SMC protein expression	↑	↑	↑↑
vWF expression	none	none	↑
Progerin expression	none	↓	↓
ECM deposition	↓	none	↓
Inflammation	↓	none	↓
Calcification	↓↓	↓	↓↓↓
Cell Density	↑	none	↑
